# Prokaryotic and Eukaryotic Community Structure in Field and Cultured Microbialites from the Alkaline Lake Alchichica (Mexico)

**DOI:** 10.1371/journal.pone.0028767

**Published:** 2011-12-14

**Authors:** Estelle Couradeau, Karim Benzerara, David Moreira, Emmanuelle Gérard, Józef Kaźmierczak, Rosaluz Tavera, Purificación López-García

**Affiliations:** 1 Unité d'Ecologie, Systématique et Evolution, CNRS UMR 8079, Université Paris-Sud, Orsay, France; 2 Institut de Minéralogie et de Physique des Milieux Condensés, CNRS UMR 7590, Université Pierre et Marie Curie, Paris, France; 3 Institut de Physique du Globe de Paris, CNRS UMR 7154, Université Paris Diderot, Paris, France; 4 Institute of Paleobiology, Polish Academy of Sciences, Warszawa, Poland; 5 Departamento de Ecología y Recursos Naturales, Facultad de Ciencias, Universidad Nacional Autónoma de México, Distrito Federal, Mexico; Argonne National Laboratory, United States of America

## Abstract

The geomicrobiology of crater lake microbialites remains largely unknown despite their evolutionary interest due to their resemblance to some Archaean analogs in the dominance of in situ carbonate precipitation over accretion. Here, we studied the diversity of archaea, bacteria and protists in microbialites of the alkaline Lake Alchichica from both field samples collected along a depth gradient (0–14 m depth) and long-term-maintained laboratory aquaria. Using small subunit (SSU) rRNA gene libraries and fingerprinting methods, we detected a wide diversity of bacteria and protists contrasting with a minor fraction of archaea. Oxygenic photosynthesizers were dominated by cyanobacteria, green algae and diatoms. Cyanobacterial diversity varied with depth, Oscillatoriales dominating shallow and intermediate microbialites and Pleurocapsales the deepest samples. The early-branching Gloeobacterales represented significant proportions in aquaria microbialites. Anoxygenic photosynthesizers were also diverse, comprising members of Alphaproteobacteria and Chloroflexi. Although photosynthetic microorganisms dominated in biomass, heterotrophic lineages were more diverse. We detected members of up to 21 bacterial phyla or candidate divisions, including lineages possibly involved in microbialite formation, such as sulfate-reducing Deltaproteobacteria but also Firmicutes and very diverse taxa likely able to degrade complex polymeric substances, such as Planctomycetales, Bacteroidetes and Verrucomicrobia. Heterotrophic eukaryotes were dominated by Fungi (including members of the basal Rozellida or Cryptomycota), Choanoflagellida, Nucleariida, Amoebozoa, Alveolata and Stramenopiles. The diversity and relative abundance of many eukaryotic lineages suggest an unforeseen role for protists in microbialite ecology. Many lineages from lake microbialites were successfully maintained in aquaria. Interestingly, the diversity detected in aquarium microbialites was higher than in field samples, possibly due to more stable and favorable laboratory conditions. The maintenance of highly diverse natural microbialites in laboratory aquaria holds promise to study the role of different metabolisms in the formation of these structures under controlled conditions.

## Introduction

Microbialites are organosedimentary structures formed by microbially-mediated mineral precipitation and/or accretion [1]. Stromatolites are microbialites exhibiting a laminated macrofabric [2]. Their fossils are found throughout the geological record [3], [4], the oldest being 3,43 Ga old (Pilbara Craton, Western Australia) [5]. After having dominated the Precambrian, stromatolite abundance declined steeply at the onset of the Phanerozoic [6], [7]. Today, stromatolites are confined to very few marine or quasi-marine environments, such as the well-studied Shark Bay, Australia [8], [9] and Exuma Sound, Bahamas [10], [11]. Microbialites have also been described in alkaline lakes such as Lake Van, Turkey [12], [13], Pyramid Lake, USA [14], the Indonesian crater lakes Satonda [15], [16], [17] and Niuafo'ou [18], but also in the freshwater Ruidera pools [19] and the hypersaline lakes LagoVermelha, Brazil [20] and Cuatro Ciénagas, Mexico [21].

Despite their geological and evolutionary importance, the precise stromatolite formation mechanisms remain poorly understood. It has been proposed that net carbonate precipitation results from a balance between concurrent microbial metabolisms [22]. Photosynthesis (both oxygenic and anoxygenic) and sulfate reduction lead to local carbonate supersaturation, whereas heterotrophic metabolisms induce carbonate dissolution [23], [24], [25], [26]. In addition, massive cyanobacterial production of exopolymeric substances (EPS), which efficiently sequester cations such as Ca^2+^ or Mg^2+^, can also inhibit carbonate precipitation [27]. Hence, microbialite formation most likely results from the interplay between microorganisms forming complex communities and their metabolic activities under the influence of environmental conditions (e.g. photoperiod, temperature) and local chemistry (ion availability).

The characterization of microbial diversity is thus crucial to further understand microbe-mineral interactions in microbialites. Most diversity studies using molecular methods have focused on marine stromatolites, where Alpha- and Gammaproteobacteria, Cyanobacteria and Planctomycetales appear to dominate [28], [29], [30], [31], [32], [33], [34], [35]. In contrast, knowledge about lacustrine microbialites remains much sparser. Firmicutes, Gamma- and Alphaproteobacteria were the most abundant taxa in Lake Van microbialites, but these studies were carried out on 15 year-old dry samples and, hence, probably biased [13]. Recent metagenomic analysis of Cuatro Ciénagas microbialites revealed a complex community where Cyanobacteria, Alpha- and Gammaproteobacteria and Planctomycetales predominated, as in marine microbialites, identifying functions potentially linked to complex redox-dependent activities and the establishment of structured biofilms [21]. Despite these pioneering studies, the precise role in mineralization and biofilm dynamics of many bacterial taxa, but also of the much less studied eukaryotic and archaeal communities, remains to be elucidated.

Understanding the role of microorganisms in stromatolite formation and the environmental conditions promoting it requires extending microbial diversity studies to other systems, including non-hypersaline or freshwater microbialites. Indeed, lacustrine microbialites may be better analogs for several Archaean stromatolites. The fossil 3.5 Ga-old Australian stromatolites likely formed in a caldera lake [36] and the exceptionally preserved 2,7 Ga-old massive stromatolites from Tumbiana also grew under lacustrine conditions [37], [38], [39]. The alkaline (pH∼8.9) Alchichica crater lake in the Central Mexico Plateau is particularly interesting from this perspective. Located at 2300 m above sea level and with a maximum depth of 63 m, it harbors prominent living microbialites down to at least 14 m deep [40]. Conspicuous dry microbialites emerge on the shores due to the 3–5 m lowering of the water level in the past three decades [41]. Alchichica is a monomictic lake, i.e. stratified during most of the year, the oxygenated surface water mixing with deep anoxic water only during the winter season [42]. Hydrochemistry studies show that water is Mg-rich (Mg/Ca = 40), oversaturated with magnesium and calcium carbonates [40], [43]. Accordingly, Alchichica microbialites are predominantly composed of hydromagnesite [Mg_5_(CO_3_)_4_(OH)_2_.4(H_2_O)] [40].

Classical morphological observations and preliminary molecular analyses focused on cyanobacteria suggested that Oscillatoriales and Pleurocapsales dominate these microbialites [40]. Here, we applied cultivation-independent molecular approaches to (i) characterize the diversity of microorganisms of the three domains of life, Bacteria, Archaea and Eucarya, in Alchichica microbialites along a 0–14 m depth gradient, (ii) compare the microbial community structure in lake microbialites with that of Alchichica microbialites maintained for two years under controlled laboratory conditions and (iii) identify microbial taxa potentially involved in carbonate precipitation and microbialite formation.

## Results

### Microbial community fingerprinting analyses of field and aquarium Alchichica microbialites

**Figure 1:**
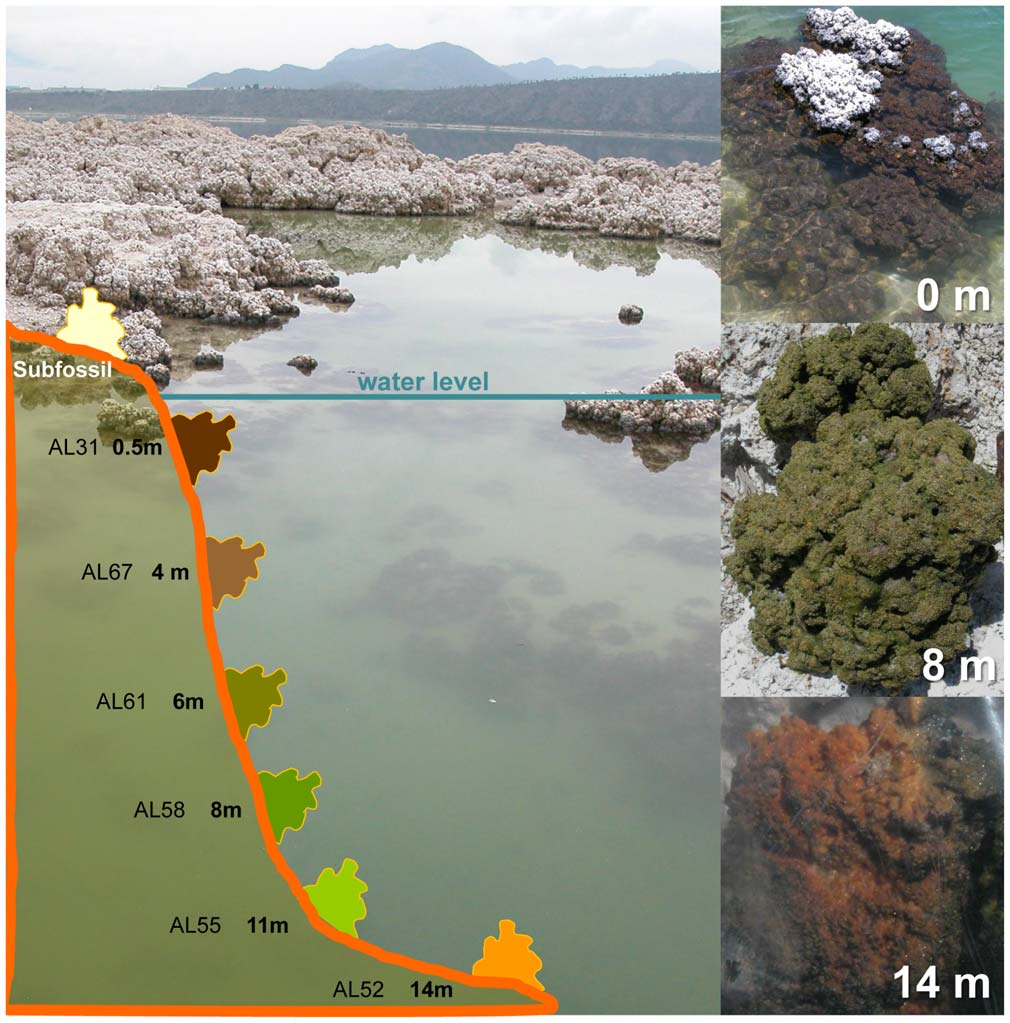
View of Alchichica and schematic depth profile showing the different sampling depths in the lake. Stromatolite fragments from three different depths and colors are shown on the right.

**Figure 2:**
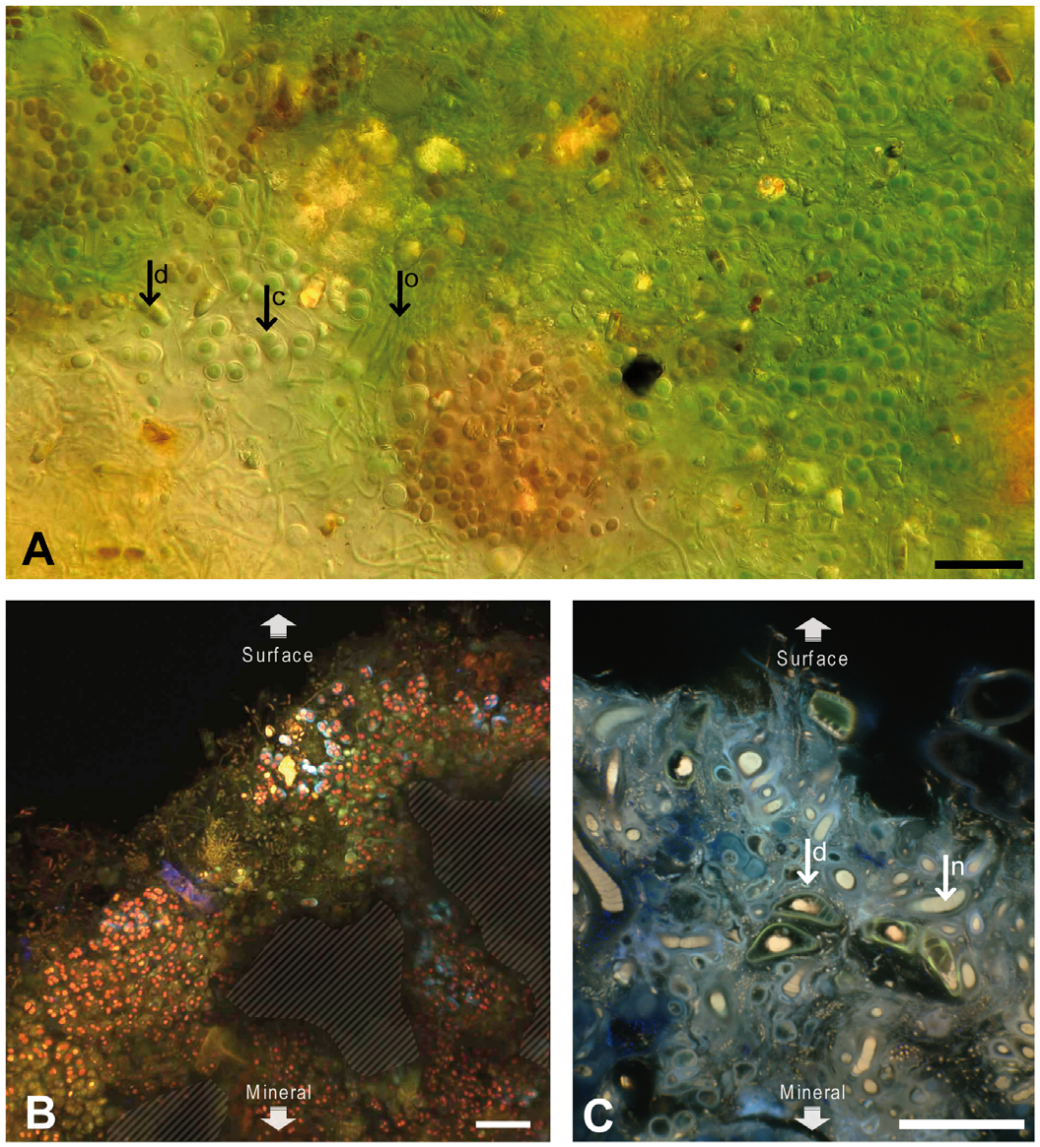
Images of biofilms associated to Alchichica microbialites. (A) Photomicrograph of a fresh biofilm associated with AQ2 microbialite showing the abundance and diversity of Cyanobacteria. (B) and (C) Natural fluorescence CLSM pictures of transversal sections of AQ2 and AL66 (4 m) microbialite surfaces, respectively. AL66 (C) was stained with DAPI and calcein. Mineral areas are indicated by stripes. Biofilm biomass was dominated by photosynthetic organisms, mostly cyanobacteria of different orders, but also diatoms and green algae. Some distinguishable morphotypes are highlighted; d, diatom; c, Chrooccocales; o, Oscillatoriales; n, Nostocales. Scale bars, 20 µm.

Field microbialites exhibited different colors depending on the sampling depth (Table 1). Sub-fossil microbialites at the rim of the lake, out of the water, were predominantly white. Submerged, living microbialites close to the surface were dark brown to black, those at 6–8 m depth intensely emerald-green and those at the highest depth sampled (14 m) golden-brown (Figure 1). This suggests that the dominant associated communities and/or their photosynthetic and protective pigments vary according to light intensity. These differences in color were also visible in the samples set on the aquaria soon after collection (Figure S1), though they disappeared with time and, after one year, all microbialite fragments in aquaria showed a similar green color (Figure 2A).

**Table 1:** Alchichica samples analyzed in this study.

Sample	Origin	Description
**AL29**	0,08 m	microbialite fragment, black/dark brown
AL31*	0,5 m	microbialite fragment, black/dark brown
**AL27**	0,8 m	microbialite fragment, black/dark brown
**AL43**	1 m	microbialite fragment, dark brown
**AL36**	1,5 m	microbialite fragment, dark brown
**AL38**	2 m	microbialite fragment, dark brown
**AL70**	3 m	microbialite fragment, brown
AL67*	4 m	microbialite fragment, brown/dark green
**AL64**	5 m	microbialite fragment, dark green
**AL61**	6 m	microbialite fragment, green
**AL58**	8 m	microbialite fragment, intense emerald green
**AL55**	11 m	microbialite fragment, intense green/yellowish
AL52*	14 m	microbialite fragment, golden/brownish
AQ1*	Aquarium 1	microbialite fragment
**AQ1b**	Aquarium 1	aquarium glass wall biofilm
**AQ1w**	Aquarium 1	water sample
AQ2*	Aquarium 2	microbialite fragment
**AQ2b**	Aquarium 2	aquarium glass wall biofilm
**AQ2w**	Aquarium 2	water sample

Samples used for clone library construction are noted with an asterisk. AQ1, aquarium 1; AQ2, aquarium 2.

To rapidly evaluate the complexity of the microbial communities in these microbialites and select representative samples for in-depth analyses, we obtained bacterial denaturing gel gradient electrophoresis (DGGE) fingerprints of 13 samples from different lake depths plus samples from the two aquaria (Figure S2). Cluster analysis of DGGE profiles divided the samples in two major groups. One corresponded to shallow samples (0.5–2 m), whereas the second included deeper (3–14 m) and aquarium samples. This was consistent with the fact that the aquarium fragments analyzed (Figure S1) corresponded originally to 3 m (AQ1) and 6 m (AQ2) depth and suggested that, at least partly, the native bacterial community was maintained in culture. Fingerprints from deeper samples displayed more bands, reflecting either a higher bacterial diversity or the fact that a few phylotypes dominate surface microbialites, masking minor components. The identity of some dominant and characteristic bands was investigated subsequently.

Based on DGGE profiles, we selected the three samples AL31 (0.5 m), AL67 (4 m) and AL52 (14 m), which displayed characteristic profiles and grouped in different clusters (Figure S2). More importantly, they were well distributed along the depth gradient and represented three phenotypic types in terms of color (Figure 1).

### Overview of bacterial diversity in Alchichica microbialites

**Figure 3:**
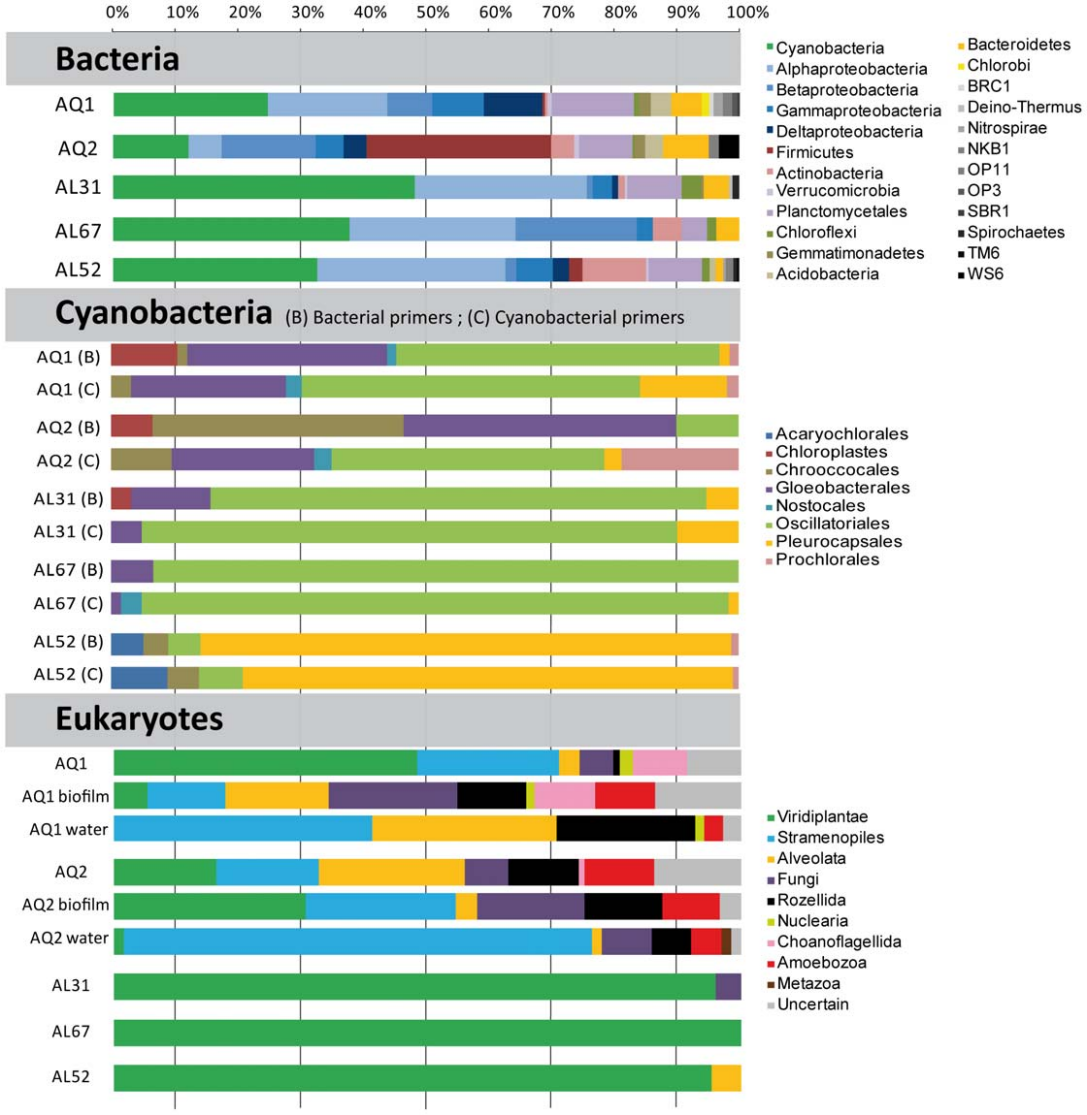
Phylogenetic distribution of bacterial, cyanobacterial and eukaryotic SSU rRNA gene sequences in Alchichica microbialites. In the specific panel for cyanobacteria, the phylogenetic distribution of cyanobacterial clones retrieved with universal bacterial primers (B) or with specific cyanobacterial primers (C) is shown for comparison. Sample names and origins are explained in Table 1. Non-Latin names correspond to Candidate Divisions; Deino-Thermus, *Deinococcus/Thermus* group.

Bacterial diversity in the selected samples was further characterized by SSU rRNA gene libraries (Table 2). We used general bacterial primers but also cyanobacterial-specific primers to get a finer description of the diversity within this group, since cyanobacteria usually dominate stromatolite microbial biomass, including Alchichica microbialites (Figure 2) [40], and likely play a major role in carbonate precipitation. In addition, since cyanobacterial EPS sheaths may decrease DNA extraction yield [44], [45], [46], using specific primers would help to detect underrepresented species. To further limit biases, we generated two bacterial and two cyanobacterial SSU rDNA libraries for each sample, except in cases when a single library allowed a coverage >80% and a small number of singletons (Table 2). There were only minor differences in the diversity obtained between the two libraries for each sample, mostly in relative proportions, in particular for a few cyanobacterial, alpha- and beta-proteobacterial phylotypes. The only significant difference was the presence of Firmicutes only in bacterial library 2. These differences are likely due to local heterogeneities and/or to a different coverage achieved by the libraries. However, despite these relatively minor differences, there was a rather good agreement in the bacterial diversity identified in the two libraries, which can therefore be considered as replicates. This was also the case for the most abundant cyanobacterial groups in both general and specific libraries (Figure 3). Therefore, for each sample we compiled the diversity from the two independent libraries for further inter-sample comparison.

**Table 2:** Summary of SSU rRNA gene sequences analyzed from bacterial, cyanobacterial and eukaryotic-specific gene libraries and the associated diversity indices.

	Clone libraries	No. of clones analyzed	No.of OTUs	Ace	Chao1	Chao1 95% confidence interval	singletons	Coverage (%)
**Bacteria**	AQ1 Library 1	84	56	198	169	104/323	43	49
	AQ1 Library 2	192	93	243	215	153/339	61	68
	AQ1 total (1+2)	276	126	423	313	228/468	87	68
	AQ2 Library 1	65	42	181	147	81/328	33	49
	AQ2 Library 2	200	57	82	74	63/103	30	85
	AQ2 total (1+2)	265	86	149	134	108/190	48	82
	AL31 Library 2	199	53	119	131	82/260	31	84
	AL67 Library 2	202	31	43	42	34/73	12	94
	AL52 Library 1	44	17	39	35	21/92	11	75
	AL52 Library 2	196	67	137	113	88/171	39	80
	AL52 total (1+2)	240	74	137	122	95/180	41	83
**Cyanobacteria**	AQ1 Library 1	53	9	10	12	9/34	3	94
	AQ1 Library 2	108	7	7	7	/	0	100
	AQ1 total (1+2)	161	16	16	16	/	1	99
	AQ2 Library 1	49	13	20	21	15/56	5	90
	AQ2 Library 2	101	19	30	28	20/64	8	92
	AQ2 total (1+2)	150	22	29	36	25/89	8	95
	AL31 Library 2	63	8	11	9	8/23	3	95
	AL67 Library 2	62	6	8	6	6/14	1	98
	AL52 Library 1	39	5	5	5	/	1	97
	AL52 Library 2	61	8	10	9	7/22	3	95
	AL52 total (1+2)	100	11	21	26	14/79	6	94
**Eukaryotes**	AQ1	95	21	32	28	22/53	9	91
	AQ1 b	76	22	24	23	22/31	4	95
	AQ1 w	69	19	74	31	21/74	12	83
	AQ2	117	23	30	27	23/45	7	94
	AQ2 b	83	16	16	16	16/19	2	98
	AQ2 w	72	11	20	21	13/63	5	93
	AL31	48	1	0	1	1/1	0	100
	AL67	38	1	0	1	1/1	0	100
	AL52	38	5	7	6	6/14	2	95

AQ1b and AQ2b refer to aquarium wall-attached biofilm samples; AQ1w and AQ2w refer to plankton samples.

Figure 3 shows the taxonomic distribution of bacterial clones in lake and aquarium samples. We identified members of 14 phyla and 7 candidate divisions. Remarkably, bacterial diversity was generally higher in aquaria than in field samples in terms of high-rank taxa, in agreement with the DGGE analysis, which showed more bands in the aquarium profiles (Figure S2). At phylum level, AQ1 taxa resembled those of field microbialites, especially those collected at higher depth. They were all dominated by Cyanobacteria and the Alpha subdivision of the Proteobacteria (60 to 75% of the total bacterial SSU rDNAs in field sample libraries, Figure 3). AQ2 displayed similar taxonomic composition, but Cyanobacteria and Alphaproteobacteria accounted for only ∼15% of sequences. In contrast, Firmicutes, minor components in the other libraries (0 to 2%), were dominant in AQ2 (29%). The rest of bacterial taxa had variable relative proportions, probably reflecting local spatial heterogeneities and/or depth-related adaptation. For example, Betaproteobacteria represented 19% of sequences in AL67 but less than 2% in other samples. The proportion of Actinobacteria increased with depth (from 1% to 10%) whereas Bacteroidetes showed the opposite trend (from 4% to 1%). Planctomycetales was one of the most constant and abundant phyla with nearly 10% of sequences in all samples, except AL67 (only 4%). AQ1 contained larger proportions of Gammaproteobacteria (8%), Deltaproteobacteria (9%) and Planctomycetales (13%) than lake samples. In general, the relative bacterial proportions in libraries appeared distributed more evenly among phyla in the deepest sample and in aquaria samples. This was in agreement with DGGE patterns, which showed more bands in AL52, reinforcing the suggestion that diversity increased with depth (especially among heterotrophic groups).

### Cyanobacteria

Confirming microscopy observations, cyanobacteria constituted the most abundant phylum in gene libraries (Figures 2 and 3). Furthermore, the distribution among cyanobacterial orders of sequences obtained with bacterial- and cyanobacterial-specific primers was remarkably similar within each sample (Figure 3). The only exception was AQ2, with relative proportions of Chroococcales and Gloeobacterales obtained with bacterial primers much higher than those obtained with cyanobacterial primers, dominated by Oscillatoriales.

Considering all samples together, we retrieved OTUs belonging to 7 of the 8 described cyanobacterial orders (only Stigonematales were absent). The most remarkable observation was the shift of relative abundance of Oscillatoriales with depth. They largely dominated surface and intermediate microbialite sample libraries (∼80% in AL31 and 90% in AL67), whereas Pleurocapsales dominated deep microbialite libraries (∼80% of cyanobacterial sequences in AL52, Figure 3). In addition, Gloeobacterales were also very abundant, especially in aquarium samples (20–40% of cyanobacterial sequences, Figure 3). Chroococcales, Nostocales and Prochlorales were detected in low proportions in all samples, whereas Acaryochlorales were exclusively amplified from the deepest sample, AL52.

**Figure 4:**
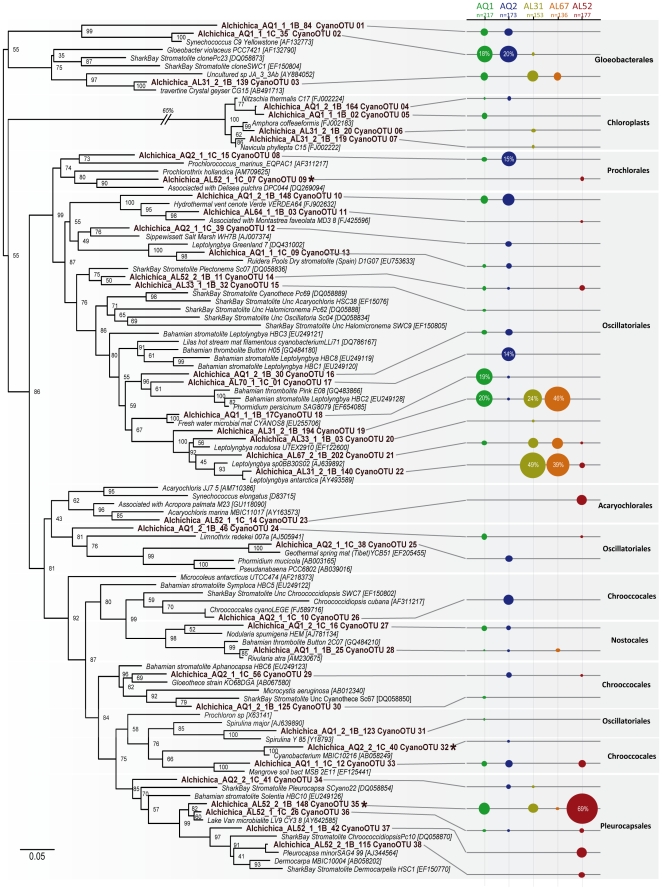
Maximum likelihood (ML) phylogenetic tree of SSU rDNA of cyanobacteria and chloroplasts from Alchichica microbialites. Numbers at nodes indicate bootstrap values. Sequences from this study are in bold. Relative proportions of the different OTUs in each sample are indicated by circles of proportional size on the right. The number (n) indicates the number total of clones analyzed for each sample. Asterisks indicate OTUs also identified in DGGE patterns. The scale bar indicates the number of substitutions per site for a unit branch length.

At a finer phylogenetic scale, we detected 38 cyanobacterial OTUs (including 4 diatom chloroplast sequences): 9 OTUs only in lake samples, 17 only in aquaria, and the remaining 12 were shared (Figure 4). OTU diversity was thus larger in aquaria microbialites compared to field microbialites. Oscillatoriales were the most diverse group with 16 OTUs, including 3 of the most abundant ones. These affiliated to the genus *Leptolyngbya* and were also detected in AL31 and AL67. Pleurocapsales were the second most diverse group with 5 OTUs. One of them (CyanoOTU35) accounted for 69% of all cyanobacterial sequences in the 14 m-deep sample AL52 (Figure 4). This phylotype was also present in the other lake samples and in AQ1, though in lower proportions. Its high abundance in deep samples was corroborated by DGGE analyses, corresponding to one of the most intense bands in deep sample fingerprints (band J in samples AL58, AL55 and AL52, Figure S2 and Table 3).

**Table 3:** Closest Alchichica microbialite OTUs to sequences of DNA fragments amplified from DGGE bands.

Band	First hit	Identity	Taxonomy	Corresponding OTU
**A**	Contig_AL67_2_1B_154	84%	Bacteria; Chloroflexi	ChlorofOTU11 *(Fig. S5)*
**B**	Contig_AL67_2_1B_105	98%	Bacteria; Chloroflexi	ChlorofOTU10 *(Fig. S5)*
**C**	Contig_AL31_2_B_35	100%	Bacteria; Chloroflexi	ChlorofOTU02 *(Fig. S5)*
**D**	Contig_AL67_2_1B_187	86%	Bacteria; Chloroflexi	ChlotofOTU01 *(Fig. S5)*
**E**	Contig_AL67_2_1B_14	91%	Bacteria; Bacteroidetes	BactOTU10 *(Fig. S9)*
**F**	Contig_AQ2_2_1B_199	97%	Bacteria; Bacteroidetes	BactOTU07 *(Fig. S9)*
**G**	Contig_AL31_2_1B_35	100%	Bacteria; Chloroflexi	ChlorofOTU02 *(Fig. S5)*
**H**	Contig_AL67_2_1B_14	98%	Bacteria; Bacteroidetes	BactOTU10 *(Fig. S9)*
**I**	Contig_AQ2_2_1C_40	97%	Bacteria; Cyanobacteria; Chroccocales	CyanoOTU32 *(* *Fig. 4* *)*
**J**	Contig_AL52_1_1C_37	97%	Bacteria, Cyanobacteria, Pleurocapsales	CyanoOTU35 *(* *Fig. 4* *)*
**K**	Contig_AQ1_1_1B_10	99%	Bacteria; Chloroflexi	ChlorofOTU07 *(Fig. S5)*
**L**	Contig_AL52_1_1C_07	91%	Bacteria; Cyanobacteria; Prochlorales	CyanoOTU09 *(* *Fig. 4* *)*
**M**	Contig_AL31_2_1B_35	100%	Bacteria; Chloroflexi	ChlorofOTU02 *(Fig. S5)*
**N**	Contig_AQ2_2_1B_212	98%	Bacteria; Actinobacteria; Rubrobacteridae	ActinoOTU03 *(Fig.S10)*

Bands correspond to those labeled in Figure S2.

In addition to Pleurocapsales, the Acaryochlorales OTU CyanoOTU23 was relatively abundant at 14 m, in agreement with the low-light-intensity adaptation characteristic of Acaryochlorales [47]. We also detected 5 Chroococcales OTUs, one of them (CyanoOTU32) particularly abundant at 8 m (AL58 sample) as shown by DGGE analyses (band I in Figure S2 and Table 3). Finally, we identified 3 very divergent OTUs belonging to the deep-branching Gloeobacterales. Among them, CyanoOTU02, identified in field sample AL31 (0.5 m), represented 18% and 20% of AQ1 and AQ2 cyanobacterial sequences.

### Other bacterial taxa with photosynthetic members

Apart from cyanobacteria, we identified phylotypes of other bacterial phyla that comprise phototrophic, in addition to heterotrophic, members: Alphaproteobacteria, Gammaproteobacteria and Chloroflexi. With ∼30% of field sample clones, Alphaproteobacteria was the second most abundant group after Cyanobacteria (Figure 3). Their relative abundance was constant with depth. They were also extremely diverse, with 68 OTUs: 35 exclusively identified in field samples, 23 in the aquaria and 10 in both field samples and aquaria (Figure S3). The composition of the deeper samples AL67 and AL52 was similar, with high proportions of Rhodospirillales and Rhodobacterales, whereas Rhizobiales were scarce in them but more abundant in the shallowest sample AL31. The most abundant Rhodobacterales OTU, AlphaOTU65 (34% and 24% of AL67 and AL52 sequences, respectively), was relatively close to members of the metabolically versatile genus *Rhodobacter*. Many *Rhodobacter* species are sulfur-oxidizing photosynthesizers and, in the context of the lake, AlphaOTU65 might actually correspond to anoxygenic photosynthesizers. Moreover, many Rhodospirillales (e.g. *Rhodospirillum*), represented by the abundant phylotypes AlphaOTU20 and AlphaOTU21, and Rhizobiales (e.g. *Rhodomicrobium*), are also anoxygenic photosynthesizers [48]. In contrast, the vast majority of Gammaproteobacteria phylotypes likely have heterotrophic metabolisms. However, some might be photosynthetic; for example the Chromatiales GammaOTU06 (Figure S4), related to environmental sequences from the Mexican alkaline lake Texcoco [49], suggesting an adaptation to these particular alkaline environmental conditions.

Chloroflexi (green non-sulfur bacteria) are typically anoxygenic photosynthesizers, although an increasing number of non-photosynthetic lineages (Anaerolineae, Caldilineae and Dehalococcoides) has also been characterized [50]. Likely phototrophic Alchichica representatives were ChlorofOTU1 and ChlorofOTU2, related to *Chloroflexus* and *Chlorothrix*, though probable heterotrophic OTUs related to *Anaerolinea* and other environmental Chloroflexi were more diverse (Figure S5). In contrast to their low proportion in gene libraries (Figure 3), DGGE analyses suggested a high abundance of Chloroflexi in Alchichica microbialites. Such difference may reflect a negative bias in the general primers used for gene library construction, as already noted in the study of Ruidera stromatolites [19], [50]. In fact, seven of the most intense DGGE bands from Alchichica field samples (bands A, B, C, D, G, K and M; Figure S2 and Table 3) were assigned to Chloroflexi after sequencing, including four (C, D, G and M) related to the two likely anoxygenic photosynthesizers ChlorofOTU1 and ChlorofOTU2. Bands C, G and M, 100% identical to the corresponding ChlorofOTU2 sequence, were detected in nearly all field samples, suggesting that this OTU was abundant at all depths. In contrast, typical photosynthetic Chloroflexi were not detected in aquaria (Figure 3).

### Typical heterotrophic bacterial taxa

Along with the potential photosynthetic OTUs mentioned above, many microbialite bacteria belonging to Alphaproteobacteria, Gammaproteobacteria (Figures S3 and S4), Chloroflexi, Chlorobi and Acidobacteria are most likely heterotrophic (Figures S5 and S6). In addition, we found 19 OTUs of Deltaproteobacteria (Figure S4), including several Myxoccocales and others corresponding most likely to sulfate-reducing bacteria (SRB). Betaproteobacteria, with 15 OTUs, were detected in all microbialite samples and particularly abundant in AL67 and AQ2 (Figures 3 and S7). The most abundant betaproteobacterial OTU in field samples (BetaOTU03) corresponded to *Delftia acidovorans*, a strict aerobe able to degrade diverse complex compounds [51].

Planctomycetales were moderately abundant (5–15% of sequences) but highly diverse, with 62 OTUs (Figure S8). Planctomycetales are able to oxidize a large range of substrates, including many different polysaccharides, which explains their frequent association to microbialites, where they probably degrade cyanobacterial EPS [13], [52]. As Planctomycetales, Bacteroidetes were also diverse (27 OTUs, Figure S9). They are known to oxidize complex organics like cell wall polymers [53].

We also identified Gram positive bacteria. Firmicutes were relatively diverse (18 OTUs) but quasi-exclusively in AQ2, including several sequences related to strict fermentative anaerobes (e.g. Clostridiales) and phylotypes from anoxic environments. Actinobacteria were also diverse (17 OTUs), many from AL52 (Figure S10). Some were Rubrobacterales (ActinoOTU3 specifically related to *Rubrobacter radiotolerans*), known for their high resistance to UV and ionizing radiation [54]. This could reflect the fact that Alchichica is at high altitude and, therefore, exposed to strong UV radiation.

Although most Chlorobi (green sulfur bacteria) are photosynthetic [48], the only Alchichica OTU from this group was related to the chemoheterotroph *Ignavibacterium album* (Figure S5). Likewise, a phototrophic lifestyle could not be predicted for the Acidobacteria sequences (Figure S6), very distantly related to the photoheterotroph *Chloracidobacterium* [55]. Finally, we identified bacteria belonging to eleven additional phyla or candidate divisions: Verrucomicrobia, Spirochaeta, with several OTUs related to sequences detected in alkaline or hypersaline microbialites and microbial mats, the nitrite-oxidizing Nitrospira, Thermus/Deinococcus, and the candidate divisions OP11, WS6, SBR1, BRC1, NKB19, TM6 and OP3 (Figures S8 and S11).

### Archaeal diversity

Despite of the use of different archaeal-specific primers and PCR conditions, we failed to amplify archaeal sequences from field samples selected for detailed study (AL31, AL67, AL52). From the rest of samples, we only retrieved two archaeal phylotypes from AL70 (3 m) and the aquarium sample AQ1 (Figure S12 and Table 1). ArchaeOTU01 was a singleton related to euryarchaeotal hot spring or hypersaline mat environmental clones. ArchaeaOTU02, detected in both AL70 and AQ1, was close to the Thaumarchaeota *Cenarchaeum* and *Nitrosopumilus* and, thus, probably an ammonium-oxidizer [56]. These results suggest that archaea are present in the microbialites but in minor proportions and very low diversity.

### Protist diversity

Although protists are conspicuous microbialite inhabitants [57], their diversity in these environments has been rarely studied. To prevent library saturation with animal sequences, we amplified SSU rRNA genes using the primer UNonMet, biased towards non-metazoan eukaryotes [58]. In addition to libraries from the selected samples AL31, AL67, AL52 and aquarium microbialites, we amplified protist SSU rDNAs from the aquarium plankton (AQ1w and AQ2w) and non-calcified biofilms growing on aquarium walls (AQ1b and AQ2b). These samples should serve as controls to identify specific protist phylotypes associated with growing microbialites. The number of clones analyzed for each sample is summarized in Table 2.

**Figure 5:**
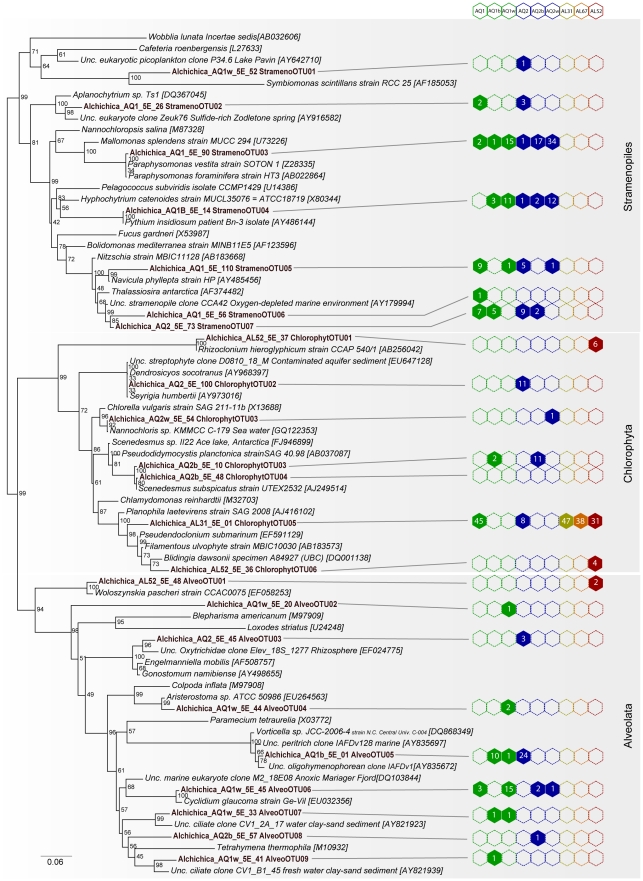
ML phylogenetic tree of bikont eukaryotic SSU rDNA sequences from Alchichica microbialites. Numbers at nodes indicate bootstrap values. Sequences from this study are in bold. Numbers of clones retrieved from each sample for each OTU are given on the right. The scale bar indicates the number of substitutions per site for a unit branch length.

There were important differences between field and aquarium samples and also between plankton and microbialites in the aquaria, whereas the aquarium non-calcified biofilms were similar to the aquarium microbialites (Figure 3). Field microbialites were dominated by one single chlorophyte (ChlorophytOTU05, related to the sessile genera *Pseudendoclonium* and *Blidingia*), representing ∼90% of all sequences in AL31 and AL67, and ∼70% in AL52 (Figure 5). Two additional chlorophytes were identified in AL52: ChlorophytOTU06, also related to those two genera, and ChlorophytOTU01, very close to *Rhizoclonium hieroglyphicum*, an entangling filamentous algae widespread in microbial mats in fresh or brackish waters [59]. AL52 also contained a dinoflagellate OTU related to the photosynthetic genus *Woloszynskia*. No other photosynthetic eukaryotes were found in the lake, although they certainly exist since living diatoms were observed by microscopy (Figure 2) and their chloroplast SSU rRNA genes were detected in sample AL31 (see above). Field samples were thus dominated by green algae, which possibly masked other eukaryotes present in minor proportions. Thus, only two additional non-photosynthetic phylotypes were identified in AL31, both corresponding to fungi (Figure S13).

Aquaria samples were far more diverse. Among photosynthetic protists, ChlorophytOTU05, dominant in field microbialites, was also abundant in aquarium microbialites, especially AQ1. However, it was absent from both the aquarium plankton and the non-calcified biofilms (Figure 5). It thus seems specifically associated to microbialites, opening the possibility that it plays a role in their formation or stability. A few other chlorophytes and several other photosynthetic lineages were identified in aquaria, notably diatoms (StramenoOTU05-07) and chrysophytes (StramenoOTU03, frequent in plankton). Concerning heterotrophic eukaryotes, ciliates (Figure 5) and very diverse opisthokonts were found in the aquaria (Figure S13). The latter included most notably Fungi, with typical Ascomycota, Basidiomycota and Chytridiomycota, but also OTUs of the environmental LKM11 group, now classified as Rozellida or Cryptomycota [60]. A relatively large diversity of Amoebozoa and choanoflagellates was also found, the latter almost exclusively in AQ1 and never in the planktonic fraction. We also identified nucleariids and several divergent sequences at the base of the Choanoflagellida/Icthyosporea and at the base of the Metazoa without close relatives (Figure S13).

## Discussion

To address the long-term question of understanding microbial-mineral interactions and how microbialites form, we first aimed at characterizing microbial communities inhabiting Alchichica microbialites at different depths. The recurrent presence of particular abundant lineages may point out to specific metabolisms and lead to hypotheses about their role in carbonate precipitation and microbialite formation. Another important issue is the possibility to preserve a significant fraction of the original microbial communities in laboratory aquaria. This would allow mineralization experiments under controlled conditions using complex and fairly genuine diverse microbial communities. Thus, we studied the diversity of microorganisms belonging to the three domains of life in an integrative approach rarely undertaken for this kind of systems.

### Alchichica field microbialite community structure and its variation with depth

**Figure 6:**
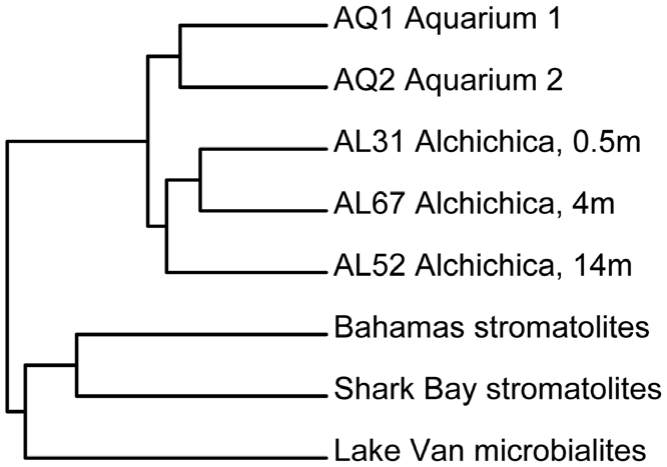
Hierarchical clustering analysis (UPGMA) of bacterial communities associated to microbialites of various settings based on pairwise UniFrac metrics. Pairwise comparisons were all significantly different (p value<0.001).

Field microbialites at all depths were largely dominated by Cyanobacteria and Alphaproteobacteria. As in Shark Bay stromatolites, where ∼10% of the Alphaproteobacteria were potential anoxygenic photosynthesizers [29], many Alchichica Alphaproteobacteria are likely photosynthetic. Most likely, Alchichica alphaproteobacterial phylotypes display diverse metabolisms going from autotrophy to heterotrophy which, together with their richness, suggests an important role in microbialite biofilm organization and activity. Chloroflexi, present in all samples and probably abundant according to DGGE fingerprinting, was the third Alchichica bacterial group with photosynthetic members. In addition to photosynthesizers, typical heterotrophs such as Planctomycetales, Bacteroidetes and Actinobacteria, were recurrently present at relative high frequency, whereas Beta-, Gamma- and Deltaproteobacteria and Firmicutes showed more variable proportions (Figure 3). The dominant Cyanobacteria and Alphaproteobacteria, accompanied by relatively abundant Planctomycetales, Firmicutes and Bacteroidetes have been reported in comparable systems including Cuatro Ciénagas [21], Bahamas [34], [35] and Shark Bay [29], [33]. In addition, many of the closest relatives to Alchichica sequences come from alkaline systems, notably the giant microbialites of Lake Van, more similar by its physico-chemical characteristics to Alchichica microbialites than marine or hypersaline lake ones [13]. This observation was statistically confirmed by comparing the bacterial community composition of Alchichica samples with those of Shark Bay, Bahamas and Lake Van. All Alchichica samples clustered together, forming two clusters, one for lake samples, with 0.5 and 4 m depth samples more closely related, and the other for aquarium microbialites (Figure 6). From the other samples, although much more distant, Lake Van was closer to Alchichica samples than the marine stromatolites.

Two important observations can be outlined from Alchichica microbialite bacterial diversity. First, even if many photosynthetic lineages are present, the relative abundance of typical heterotrophic lineages suggests that they play an important role. Second, the most remarkable change along the depth gradient was the marked shift in the cyanobacterial community composition, dominated by filamentous Oscillatoriales in surface and intermediate depths (>90% of sequences at 0.5 and 4 m) and by Pleurocapsales in deeper samples (>80% of sequences, contributed mostly by the phylotype CyanoOTU35). This shift was detected by gene library comparison but also by sequencing intense DGGE bands (Figure S2 and Table 3). Although variation of the cyanobacterial composition at larger spatial scales (>few centimeters), as evidenced in Hamelin Pool [29], [33] and Bahamas [32], cannot be discarded, the Oscillatoriales-to-Pleurocapsales dominance transition with depth in Alchichica is likely related to adaptation to depth and light intensity. Oscillatoriales are indeed adapted to high light intensity [61], whereas Pleurocapsales actively search low light (Waterbury and Stanier, 1978). This correlates with microscopy observations showing that filamentous Oscillatoriales tend to grow at the microbialite surface (e.g. Figure 2A), where their massive presence can obscure that of other microbialite-associated bacteria, whereas the coccoid or pseudo-filamentous Pleurocapsales are intimately associated to the mineral matrix (unpublished observations). The differential presence of these morphologically dissimilar cyanobacteria may have a significant impact on the organization of the microbialite biofilms.

In contrast with the recognized importance of bacteria in microbialite formation and dynamics, archaea and microbial eukaryotes might have been overlooked in the past. Our results show that archaea are present in Alchichica microbialites but in minor proportions and very low diversity. This confirms observations from Shark Bay (7% of sequences, [29], Bahamas (1–2%, [31] and Cuatro Ciénagas [21] stromatolites. Consequently, the role of archaea in microbialite formation is probably minor. Inversely, some reports based on microscopy observations suggest that microbial eukaryotes could be relevant in microbialites [57], [62], though their diversity has rarely been assessed. The only available molecular studies, done in Shark Bay and Bahamas stromatolites [34], detected a very low diversity of eukaryotes compared to bacteria. However, protist diversity might have been underestimated with general eukaryotic primers, which lead to rapid saturation of gene libraries by metazoan sequences, especially nematodes [34]. Thus, very little is known about protist contribution to biofilm biodiversity, biomass, structure and lithification. Here, we avoided gene library saturation by metazoans using primers biased against animal sequences. Despite so, we found a low eukaryotic diversity in field microbialites, corresponding essentially to chlorophytes, with some fungi in the deepest sample. Therefore, the major eukaryotic players in Alchichica appear to be green algae, with a same phylotype (ChlorophytOTU05) dominating along the depth gradient.

Finally, although a variety of physico-chemical parameters were measured in the water column during sampling as well as subsequent microbialite mineralogical and isotopic analyses [40], establishing correlations of these with microbialite microbial community composition is difficult because of the inherent heterogeneity of these systems. Microbialites are irregular, exhibiting different orientations to light at a same depth, and are spatially structured, offering a variety of niches with different physico-chemical parameters at microscale. Establishing meaningful correlations between local environmental parameters and microbial diversity will require further studies at microscale.

### Field versus aquaria microbialites

The observation of a large microbial diversity in microbialites maintained for two years in the laboratory was unexpected for two reasons. First, only relatively small microbialite fragments were installed in aquaria, which might not carry individuals of all the microbial species living in the lake microbialites. Second, since the laboratory conditions were much more stable (e.g., a remarkably constant pH, Figure S1), we expected that a few, perhaps opportunistic, lineages became dominant and excluded the rest of the native microbial diversity. However, not only the diversity of most of the abundant lineages found in the lake was maintained, but bacteria and eukaryotes were much more diverse in laboratory microbialites (Table 2). Indeed, bacterial communities in aquaria were, despite their differences, more similar between them than to the lake samples (Figure 6).

The increase of microbial diversity in aquaria concerns very diverse groups thriving at pH 8.9. This minimizes the possibility of potential contaminants coming from the laboratory, which would be outcompeted by the well-adapted Alchichica alkaliphiles. The stable conditions in aquaria appear not only to have maintained organisms dominant in the different field samples, but also favored the growth of microbes that were in low proportions in the lake. To our knowledge, there is only another example that compares the diversity of cultured versus natural microbialites [28]. Although in this case cultured microbialites were artificial (fused oolitic sand grains inoculated with Bahamian stromatolite microorganisms), a good preservation of the community composition after 1.5 years was inferred by comparison of Shannon indices. These observations suggest a remarkable resilience of microbialite communities.

Gloeobacterales offer an example of increased diversity and abundance in aquaria microbialites (Figure 3). These cyanobacteria have raised much attention because of their basal position in phylogenetic trees, being the only group branching before chloroplasts, and because unusual features such as the lack of thylakoids and particular photosystems [63], [64], [65], [66], [67]. For many years, the only cultured species was *Gloeobacter violaceous*, isolated from calcareous rock [65], [68]. More recently, “*Synechococcus* sp. C9” was isolated from a mat in Yellowstone alkaline Octopus spring [63], [69]. Several environmental sequences were recently added to the group, mostly coming from microbial mats or microbialites, such as the Shark Bay stromatolites [30], [33], suggesting that the whole group may be adapted to this kind of environments. Our very distant Alchichica sequences encompass the whole known diversity within this order (Figure 4).

The larger diversity in aquarium microbialites was particularly manifest in the case of microbial eukaryotes. Whereas a few green algal phylotypes dominated lake microbialites, cultured microbialites contained those lineages but also a wide variety of other photosynthetic species, including diatoms and chrysophytes, diverse other stramenopiles and ciliates (Figure 5) and many opisthokonts (Figure S13). Possibly, most of these protists were present in the lake but throve to large, detectable amounts under stable laboratory conditions. The diversity of opisthokonts was remarkable. Several fungal lineages were detected in microbialites, notably members of the Rozellida [70] or Cryptomycota [60], which constitute the deepest lineage of fungi and groups parasitic flagellates very common in freshwater systems [71]. The diversity of choanoflagellates, amoebae, nucleariids, and several divergent sequences at the base of the Choanoflagellates/Icthyosporea and at the base of the Metazoa (Figure S13), makes these microbialites interesting to explore lineages placed at the onset of metazoan evolution.

### Microbial metabolism-based model of microbialite formation

Taking into account the most likely metabolisms of the microorganisms detected in field and aquarium microbialites, we propose to extend the model of formation of microbialites originally build on marine Bahamian stromatolites [22] to Alchichica (Figure S14). Microbialite formation would be the net result of a balance between metabolic activities favoring carbonate precipitation or dissolution, which would in turn depend on light availability (day or night) and on local physico-chemical conditions (e.g. oxic or anoxic microenvironments) [22].

As in the Bahamas case, the most important metabolism involved is probably bacterial photosynthesis, in particular the oxygenic photosynthesis carried out by the very abundant cyanobacteria. Photosynthesis drives the alkalinity engine towards carbonate precipitation by consuming bicarbonate [22] and increasing local pH [72], [73]. Although much less studied, eukaryotic photosynthesizers may play a similar role since many eukaryotic algae induce comparable changes in pH and CO_2_ concentration [74]. In addition, eukaryotic algae can provide nucleation sites [62] and trap particles [75]. Similarly, anoxygenic photosynthetic bacteria, such as the phototrophic Chloroflexi likely abundant in Alchichica, also increase local alkalinity and induce carbonate precipitation [76]. In addition, part of the H_2_S consumed by anoxygenic photosynthesis may come from the activity of SRB, represented in Alchichica by Deltaproteobacteria and Firmicutes. Sulfate reduction generates carbonate ions, thus being another activity potentially leading to carbonate precipitation [23], [24], [25], [77]. This process, independent of light availability, can take place during day and night.

Alchichica microbialites also contain diverse and abundant heterotrophic bacteria, including Planctomycetales, Bacteroidetes, Acidobacteria, many Proteobacteria and various others. They can induce carbonate dissolution due to respiration of organic matter and production of protons [27] but they can also promote carbonate precipitation by liberating cations sequestered by EPS and other macromolecules, making them available for precipitation. Indeed, many of these heterotrophs are known to degrade complex polymeric compounds, including EPS [78]. The balance between these processes determines the net formation of carbonate.

Even if the major role in carbonate precipitation and dissolution is probably due to activities of the largely dominant bacterial community, the role of eukaryotes should not be neglected. Photosynthetic algae may have a direct role on carbonate precipitation and an indirect role associated to chemical properties of the cell walls that provide nucleation centers for crystal growth [79], [80]. In addition to their photosynthetic activity, diatoms embedded in the carbonates could be relevant for secondary silicification since, after death, their frustules supply Si. Finally, Alchichica fungi, some of which may be endolithic, also deserve further study. They could make the system more fragile by forming pervasive microborings but also serve as new calcification centers [81]. At any rate, protists play an important role as grazers and predators, exerting a control over bacteria and being involved in the fine-tuning of the community structure and its activities.

## Materials and Methods

### Sampling and maintenance of living microbialites in aquaria

Samples were collected from Lake Alchichica (N 19°25.119; W 97°23.860, Puebla State, Mexico) in July 2007. No specific permits were required for the described field studies, the location is not privately-owned or protected and the field studies did not involve endangered or protected species. Several physico-chemical parameters were measured in the water column at different depths including total dissolved solutes, pH, temperature, concentrations of Cl-, SO_4_^2−^, Br^−^, F^−^, Na^+^, Mg^2+^, K^+^, Ca^2+^, Li^+^, O_2_, Si, NO_2_^−^, NO_3_^−^, PO_4_^3−^, NH_4_^+^, N/P ratios, conductivity, alkalinity, suspended matter and the saturation index for several minerals [40]. Similarly, bulk mineralogical and isotopic (δ^13^C and δ^18^O) analyses of microbialites at different depths were carried out [40]. Living microbialite fragments were collected by scuba diving along a depth gradient from immediately below surface down to 14 m in depth. Samples for microbiology studies were picked up with gloves and sterile forceps to minimize all possible contamination, introduced in Falcon tubes and fixed in situ in ethanol (80% final concentration). They were kept at room temperature during transport, then stored at 4°C until processing. Several larger microbialite fragments (>10 cm) were placed in sterile plastic containers filled with lake water for transfer to laboratory aquaria. A layer of small (1 cm) fragments of sub-fossil, rim Alchichica microbialites was deposited at the bottom of aquaria in order to buffer the solution pH and chemical composition. Living microbialite fragments were deposited in aquarium 1 (AQ1, fragments collected at 30 cm, 3 m and 8 m depth) and aquarium 2 (AQ2, fragments collected at 80 cm, 1 m and 6 m depth). Aquaria were illuminated with 15w −10 lumens/W fluorescent tubes producing solar spectral wavelength. Photoperiods were adjusted to 12 h of daylight for AQ1 and 16 h of light for AQ2. Temperature and pH were measured once a month and water loss due to evaporation replaced by distilled water. Despite some temperature variation over time for over 3 years after collection, pH remained remarkably constant at 8.9 (Supplementary Figure S1). The aquarium microbialite samples collected for molecular analyses were taken from the fragments from 3 m (AQ1) and 6 m (AQ2) depth.

### Optical and confocal laser scanning microscopy

Fresh aquarium microbialite-associated biofilms were examined using a Zeiss Axioplan 2 optical microscope and photographed with a Canon PowerShot G5 camera. We also prepared microbialite inclusions in resin for the observation of transversal sections using confocal laser scanning microscopy (CLSM). Several samples were stained with 4′,6′-diamidino-2-phenylindole or DAPI (1 µg/ml; 10 minutes at room temperature) and/or calcein (0.1 mg/ml; 36 h at 4°C) prior to inclusion. Microbialite fragments were dehydrated in a gradual series of ethanol baths (30%, 50%, 70%, 90%, and 100%), and progressively impregnated with hard grade LR-white resin (Polysciences, Inc.). Samples were incubated for 18 h at 4°C in (1/1) then (2/1) mixture of LR-white/ethanol and finally in pure LR-white resin. After 3 h at room temperature, samples were embedded in pure LR-white resin for 1 h at 40°C and then for 24 h at 60°C. After polymerization, transverse cross-sections were cut with a diamond wire and polished (diamond powder ¼ µm). These sections were examined using a FluoViewTM FV1000 confocal laser scanning microscope with a spectral resolution of 2 nm (Olympus). The FluoViewTM FV1000 was equipped with a 405 nm laser diode, and multi-line argon (458 nm, 488 nm, and 515 nm), helium-neon-green (543 nm) and helium-neon-red (633 nm) lasers. Fluorescence images of the microbialite transversal sections were obtained with concomitant excitation at wavelengths of 405 nm, 488 nm, and 543 nm and collection of the emitted fluorescence between 425–475 nm, 500–530 nm, and 560–660 nm, respectively.

### DNA purification

Total genomic DNA was extracted 1) from ethanol-fixed field samples selected along a depth profile in the lake and 2) from aquaria microbialites 2 years after collection. A small fragment (∼1 cm^3^) from each microbialite sample was ground using a sterile agate mortar. 200 µl of the resulting powder were transferred to an eppendorf tube. Carbonates were largely dissolved by adding 100 µl of HCl at 33% for 30 s then neutralized with 1 ml of a 1∶1 mixture of PBS pH 7 and 0.5 M EDTA pH 9. Samples were centrifuged for 5 min at 12500 rpm. DNA was extracted from the pellet using two different methods. In a preliminary assay, DNA was purified with the QuickPick™gDNA Kit (Bio-Nobile, Parainen, Finland) following the instructions of the manufacturer except that samples were previously incubated for 3 h at 56°C with 0,5 µl of Proteinase K extra (20 mg/ml) and 1,5 µl of Viscozyme®. In a second assay DNA was purified using the MoBioPowerSoil DNA kit (MoBio, Carlsbad, CA, USA) after a first incubation step with 2 µl of Viscozyme® (Sigma-Aldrich, Buchs, Switzerland) (1 h at 37°C) in order to enhance degradation of the abundant exopolymeric substances. According to preliminary tests (data not shown), the second protocol produced a better extraction yield and was thus applied on every sample selected along the depth gradient. However, we include in the present study the results of libraries constructed using DNA purified with the first method as replicates. Libraries constructed using DNA purified by the first method were labeled Library 1; those made with the second one are labeled Library 2.

### Denaturing gel gradient electrophoresis (DGGE) analysis

SSU rDNA fragments of approximately 150 bp were amplified from DNA purified from different microbialite samples using the MoBio kit with the specific bacterial forward primer 341F-GCclamp (CGCCCGCCGCGCGCGGCGGGCGGGGCGGGGGCACGGGGGG CCTACGGGAGGCAGCAG) and the reverse bacterial primer 543R (ATTACCGCGGCTGCTGG) [82]. Polymerase chain reactions (PCR) were performed under the following conditions: an initial denaturation step at 94°C for 3 min, 30 cycles consisting of a denaturation step at 94°C for 15 s, an annealing step of 30 s (a touch down procedure with a decreasing annealing temperature from 65°C to 55°C for the 10 first cycles was applied followed by a hybridization temperature of 55°C for the following 20 cycles) and a polymerization step at 72°C for 1.5 min, and a final step of 1 h extension at 72°C (modified from [82]). Migration of PCR products was done in a denaturing gradient gel using the CBS Scientific (California, USA) electrophoresis system. Urea and formamide were used as denaturing agents with a concentration gradient from 30% to 60%. 50 bp-ladder markers (Promega, Lyon, France) were intercalated every three samples. The gels were stained with SYBR Gold (Invitrogen, Carlsbad, CA, USA) and photographed under UV light. Gels were normalized according to the ladder migration using the software Bionumerics® (AppliedMaths, Sint-Martens-Latem, Belgium). A distance matrix based on the presence/absence of bands in the different samples was used for cluster analysis of samples using the Jaccard coefficient [83].

### Small subunit rRNA gene library construction

We constructed SSU rDNA libraries specific for archaea, bacteria, cyanobacteria and microbial eukaryotes from five selected samples: three field samples from three different depths AL31 (0.5 m), AL67 (3 m), AL52 (14 m) and two samples from the two aquaria. Samples from aquarium microbialites used in this study were collected after 17 months (Libraries 1) and 27 months (Libraries 2). To amplify SSU rDNA, the following sets of specific primers were used: B-27F (AGAGTTTGATCCTGGCTCAG) and 1492R (GGTTACCTTGTTACGACTT) for bacteria; CYA106F (CGGACGGGTGAGTAACGCGTGA) [44] and 23S30R (CTTCGCCTCTGTGTGCCTAGGT) for cyanobacteria; Ar109 (AC(G/T)GCTGCTCAGTAACACGT) and 1492R for archaea and 82F (GAAACTGCGAATGGCTC) and UNonMet (TTTAAGTTTCAGCCTTGCG) for non-metazoan eukaryotes [58]. PCR reactions were performed under the following conditions: 30 cycles (denaturation at 94°C for 15 s, annealing at 50–55°C for 30 s, extension at 72°C for 2 min) preceded by 2 min denaturation at 94°C, and followed by 7 min extension at 72°C. Clone libraries were constructed using the TopoTA cloning kit (Invitrogen, Carlsbad, CA, USA) according to the manufacturer's instructions. Clone inserts were partially sequenced (∼800 bp) by Beckman Coulter Genomics (Takeley, United Kingdom) using first the reverse primer 1492R for bacteria (including cyanobacteria) and archaea and the forward primer 82F for eukaryotes. At least one representative clone per phylotype or Operational Taxonomic Unit (OTU, group of sequences sharing >97% identity) was fully sequenced for detailed phylogenetic analysis. Sequences were deposited in GenBank with accession numbers JN825302–JN825705.

### Phylogenetic analyses

A total of 1143 bacterial clones excluding cyanobacteria amplified with specific cyanobacterial primers, 526 cyanobacterial clones (in addition to cyanobacterial clones retrieved with general bacterial primers) and 598 eukaryotic clones were analyzed. The closest relatives to these sequences were identified by BLAST [84], [85] and retrieved from GenBank (http://ncbi.nlm.nih.gov/). Several datasets (one for each life domain and one specific for cyanobacteria) were constructed and aligned using MAFFT [86]. A preliminary phylogenetic analysis of all partial sequences was done by distance methods (neighbor-joining, NJ), allowing the identification of identical or nearly identical sequences and the selection of representative clones for subsequent analysis. The multiple alignment was then manually edited using the program ED from the MUST package [87]. Final phylogenetic trees included our sequences together with their closest relatives in GenBank and some representative cultivated species. Maximum likelihood (ML) phylogenetic trees were reconstructed using TREEFINDER [88] applying a general time reversible (GTR) model of sequence evolution, and taking among-site rate variation into account by using a four-category discrete approximation of a Γ distribution. Maximum likelihood bootstrap proportions were inferred using 1,000 replicates. Phylogenetic trees were viewed using FIGTREE [89].

### Estimates of microbial diversity and community comparison analyses

Distance matrices were generated for each clone library using ClustalX software [90]. They were used as input for the software DOTUR [91], which was used to cluster sequences in OTUs using an identity cut-off of 0.03. Richness estimations (Chao1 and Ace) were calculated using DOTUR with default settings. Coverage values were calculated using the Good estimator [92] following the equation C = (1−n/N)×100, where C is the percentage of coverage of the library, n the number of singletons and N the total number of clones examined. To compare the composition of bacterial communities associated to Alchichica microbialites with those associated to Bahamas [31] and Shark Bay [29] stromatolites as well as to the alcaline Lake Van microbialites [13], we recovered the SSU rDNA bacterial sequences from those studies and constructed an alignment containing 3040 sequences using MAFFT [86]. We then constructed an approximately maximum likelihood phylogenetic tree based on 338 unambiguously aligned positions using FastTree [93]. We then compared ß-diversity measurements and obtained pairwise p-values using UniFrac [94] as implemented in the software MOTHUR [95].

## Supporting Information

Figure S1 Alchichica microbialites maintained in laboratory aquaria. A. Initial setting of microbialites fragments in aquaria with different photoperiods. B. Microbialites after one year of cultivation in aquaria. C. Measurements of pH and temperature over time. Orange symbols (aquarium 1); blue symbols, aquarium 2. Red bars indicate points at which aquaria were sampled for the clone Library 1 and Library 2 construction of the present study.
(TIF)

Figure S2 Cluster analysis of DGGE fingerprints of bacteria associated to Alchichica microbialites. The name and depth of each sample are given on the right. AQ1 and AQ2 correspond to samples from laboratory aquaria. The scale bar above the dendrogram shows distances (%) between samples based on presence/absence of bands. Grey bars at nodes indicate the standard deviation. Bands labeled with capital letters were cut for sequencing. Samples labeled with an asterisk were chosen for detailed molecular diversity analyses.
(TIF)

Figure S3 Maximum likelihood (ML) phylogenetic tree of alphaproteobacterial SSU rDNAs from Alchichica microbialites. Numbers at nodes indicate bootstrap values. Sequences from this study are in bold. Relative proportions of the different OTUs in each sample are indicated by circles of proportional size on the right. The number (n) indicates the number total of clone analyzed for each sample. The scale bar indicates the number of substitutions per site for a unit branch length.
(TIF)

Figure S4 Maximum likelihood (ML) phylogenetic tree of SSU rDNA sequences of Deltaproteobacteria and Gammaproteobacteria from Alchichica microbialites. Numbers at nodes indicate bootstrap values. Sequences from this study are in bold. Numbers of clones retrieved from each sample for each OTU are given on the right. The scale bar indicates the number of substitutions per site for a unit branch length.
(TIF)

Figure S5 Maximum likelihood phylogenetic tree of SSU rDNA sequences of Chloroflexi and Chlorobi from Alchichica microbialites. Numbers at nodes indicate bootstrap values. Sequences from this study are in bold. Numbers of clones retrieved from each sample for each OTU are given on the right. Asterisks indicate OTUs also identified in DGGE patterns. The scale bar indicates the number of substitutions per site for a unit branch length.
(TIF)

Figure S6 Maximum likelihood (ML) phylogenetic tree of SSU rDNA sequences of Gemmatimonadetes and Acidobacteria from Alchichica microbialites. Numbers at nodes indicate bootstrap values. Sequences from this study are in bold. Numbers of clones retrieved from each sample for each OTU are given on the right. The scale bar indicates the number of substitutions per site for a unit branch length.
(TIF)

Figure S7 Maximum likelihood (ML) phylogenetic tree of SSU rDNA sequences of Betaproteobacteria from Alchichica microbialites. Numbers at nodes indicate bootstrap values. Sequences from this study are in bold. Numbers of clones retrieved from each sample for each OTU are given on the right. The scale bar indicates the number of substitutions per site for a unit branch length.
(TIF)

Figure S8 Maximum likelihood (ML) phylogenetic tree of SSU rDNA sequences of Planctomycetales and Verrucomicrobia from Alchichica microbialites. Numbers at nodes indicate bootstrap values. Sequences from this study are in bold. Numbers of clones retrieved from each sample for each OTU are given on the right. The scale bar indicates the number of substitutions per site for a unit branch length.
(TIF)

Figure S9 Maximum likelihood (ML) phylogenetic tree of SSU rDNA sequences of Bacteroidetes from Alchichica microbialites. Numbers at nodes indicate bootstrap values. Sequences from this study are in bold. Numbers of clones retrieved from each sample for each OTU are given on the right. Asterisks indicate OTUs also identified in DGGE patterns. The scale bar indicates the number of substitutions per site for a unit branch length.
(TIF)

Figure S10 Maximum likelihood (ML) phylogenetic tree of SSU rDNA sequences of Actinobacteria and Firmicutes from Alchichica microbialites. Numbers at nodes indicate bootstrap values. Sequences from this study are in bold. Numbers of clones retrieved from each sample for each OTU are given on the right. Asterisks indicate OTUs also identified in DGGE patterns. The scale bar indicates the number of substitutions per site for a unit branch length.
(TIF)

Figure S11 Maximum likelihood (ML) phylogenetic tree of SSU rDNA sequences of CD OP11, CD WS6, Deinoccocus-Thermus, CD SBR1, CD BRC1, CD NKB19, Nitrospira, CD TM6, CD OP3 and Spirochaeta from Alchichica microbialites. Numbers at nodes indicate bootstrap values. Sequences from this study are in bold. Numbers of clones retrieved from each sample for each OTU are given on the right. The scale bar indicates the number of substitutions per site for a unit branch length.
(TIF)

Figure S12 Maximum likelihood (ML) phylogenetic tree of SSU rDNA sequences of Archaea from Alchichica microbialites. Numbers at nodes indicate bootstrap values. Sequences from this study are in bold. Numbers of clones retrieved from each sample for each OTU are given in brackets. The scale bar indicates the number of substitutions per site for a unit branch length.
(TIF)

Figure S13 Maximum likelihood (ML) phylogenetic tree of SSU rDNA sequences of Unikonts (Amoebozoa plus Opisthokonta) from Alchichica microbialites. Numbers at nodes indicate bootstrap values. Numbers of clones retrieved from each sample for each OTU are given on the right. The scale bar indicates the number of substitutions per site for a unit branch length.
(TIF)

Figure S14 Hypothetical model of carbonate formation dynamics based on known metabolisms of microbial lineages detected in Alchichica microbialites. The panels represent the activities that would occur during day (left) and; night (right) in areas where oxygenic (upper panels) or anoxygenic (lower panels) photosynthesis predominates.
(TIF)
