# Supplementary figures and images for: Prokaryotic and Eukaryotic Community Structure in Field and Cultured Microbialites from the Alkaline Lake Alchichica (Mexico)
Source: PLoS One. 2011 Dec 14;6(12):e28767. doi: 10.1371/journal.pone.0028767 (PMC3237500; doi:10.1371/journal.pone.0028767)

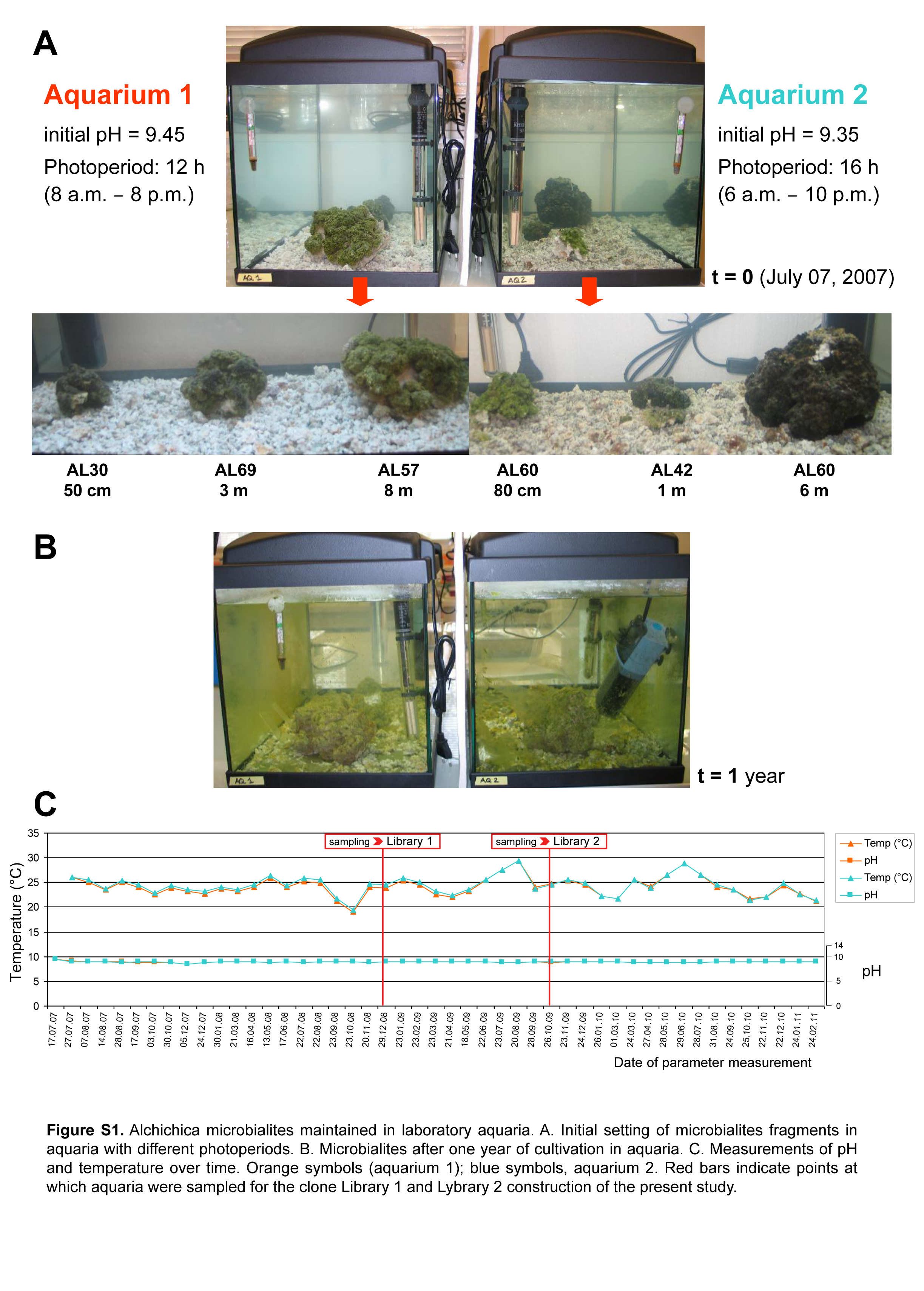

Supplement: Figure S1 — Alchichica microbialites maintained in laboratory aquaria. A. Initial setting of microbialites fragments in aquaria with different photoperiods. B. Microbialites after one year of cultivation in aquaria. C. Measurements of pH and temperature over time. Orange symbols (aquarium 1); blue symbols, aquarium 2. Red bars indicate points at which aquaria were sampled for the clone Library 1 and Library 2 construction of the present study. (TIF) [file pone.0028767.s001.tif]

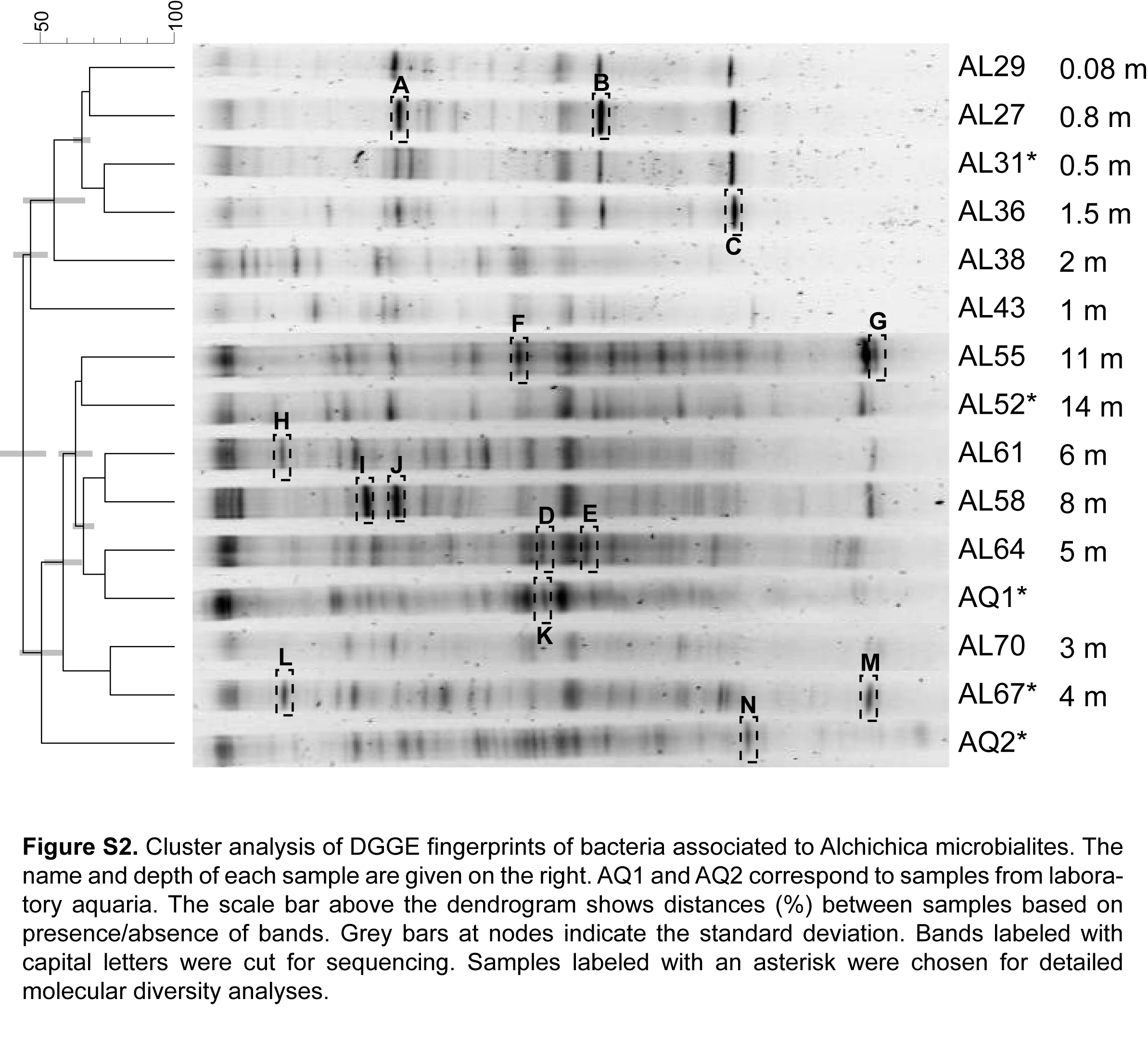

Supplement: Figure S2 — Cluster analysis of DGGE fingerprints of bacteria associated to Alchichica microbialites. The name and depth of each sample are given on the right. AQ1 and AQ2 correspond to samples from laboratory aquaria. The scale bar above the dendrogram shows distances (%) between samples based on presence/absence of bands. Grey bars at nodes indicate the standard deviation. Bands labeled with capital letters were cut for sequencing. Samples labeled with an asterisk were chosen for detailed molecular diversity analyses. (TIF) [file pone.0028767.s002.tif]

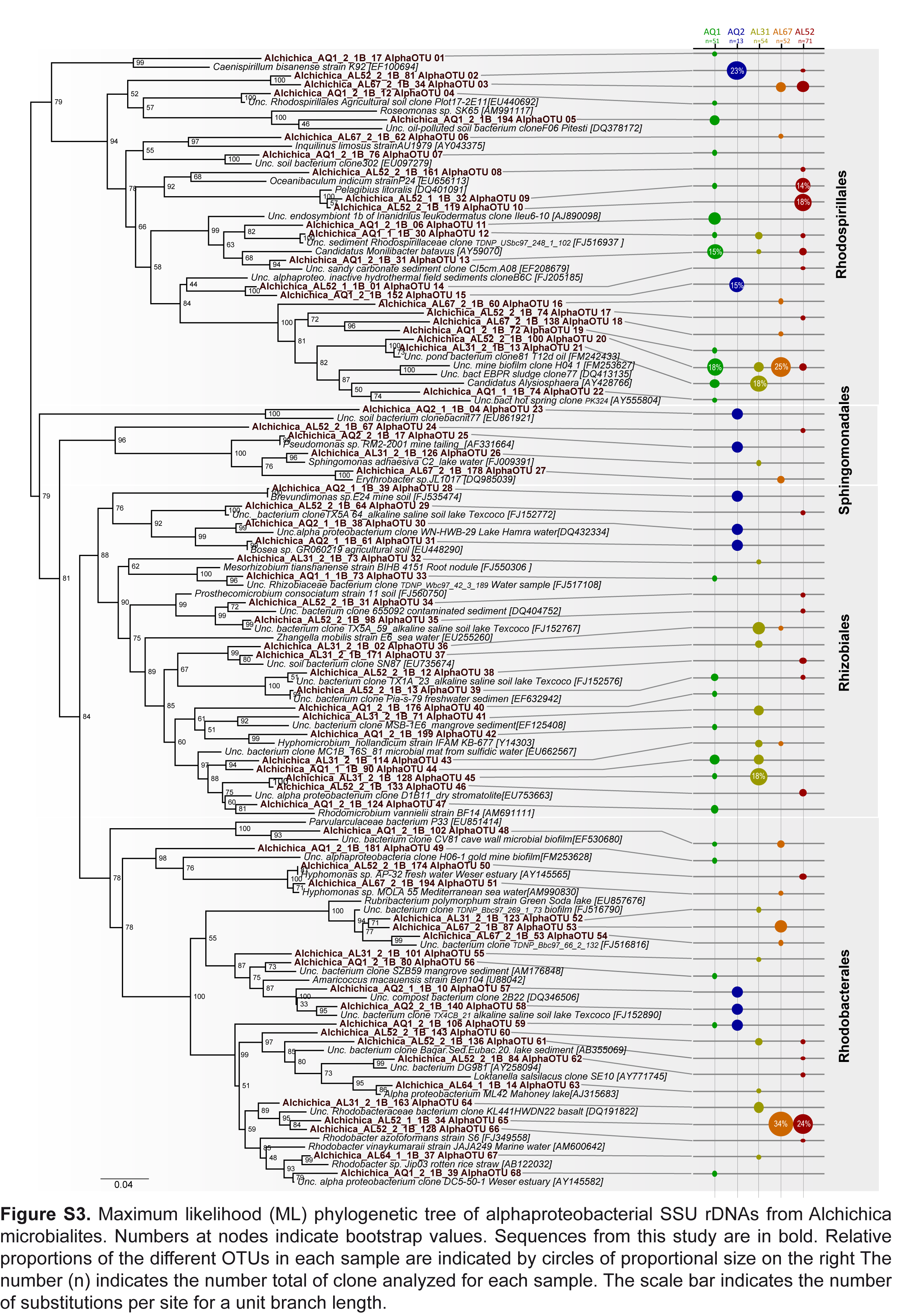

Supplement: Figure S3 — Maximum likelihood (ML) phylogenetic tree of alphaproteobacterial SSU rDNAs from Alchichica microbialites. Numbers at nodes indicate bootstrap values. Sequences from this study are in bold. Relative proportions of the different OTUs in each sample are indicated by circles of proportional size on the right. The number (n) indicates the number total of clone analyzed for each sample. The scale bar indicates the number of substitutions per site for a unit branch length. (TIF) [file pone.0028767.s003.tif]

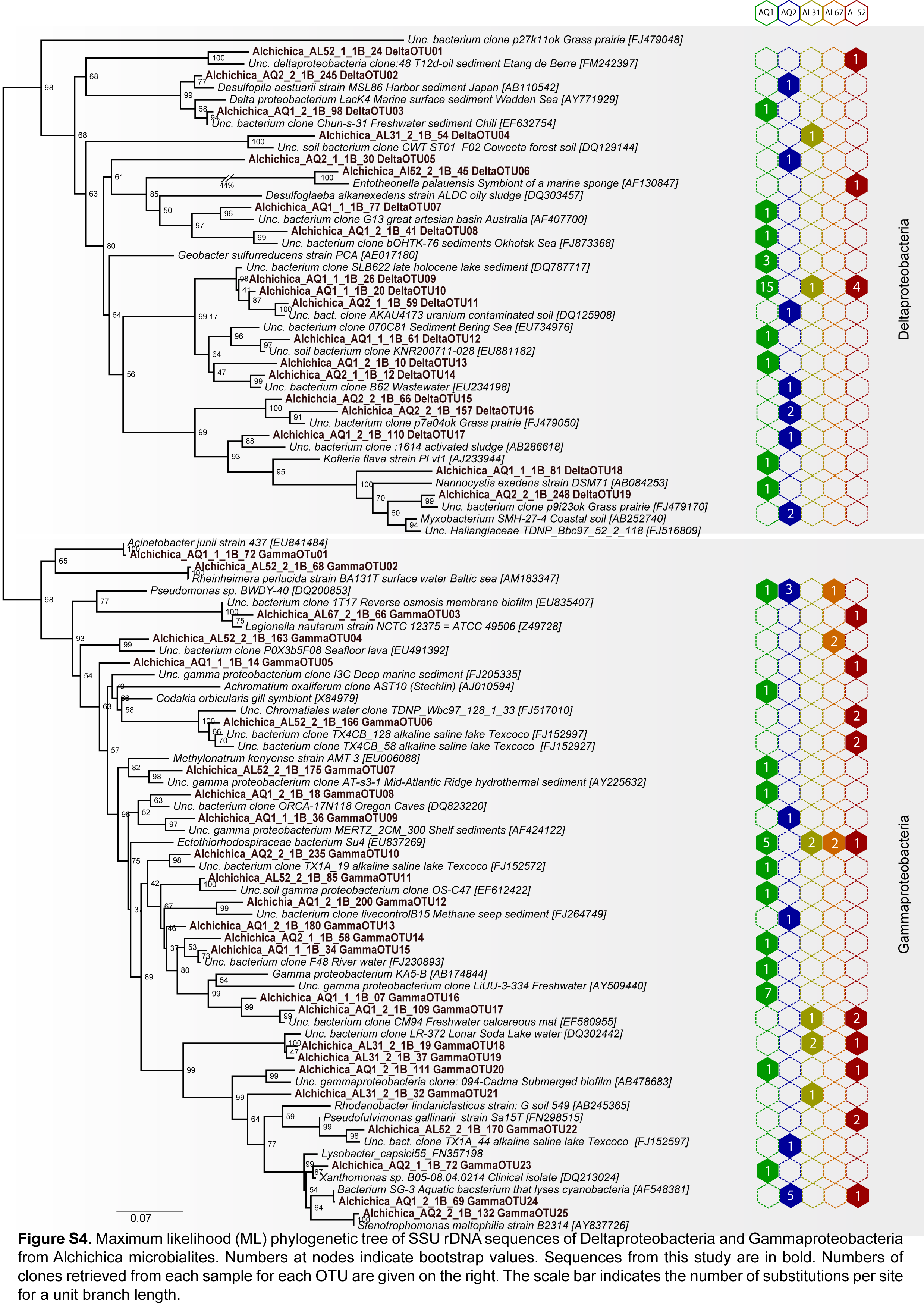

Supplement: Figure S4 — Maximum likelihood (ML) phylogenetic tree of SSU rDNA sequences of Deltaproteobacteria and Gammaproteobacteria from Alchichica microbialites. Numbers at nodes indicate bootstrap values. Sequences from this study are in bold. Numbers of clones retrieved from each sample for each OTU are given on the right. The scale bar indicates the number of substitutions per site for a unit branch length. (TIF) [file pone.0028767.s004.tif]

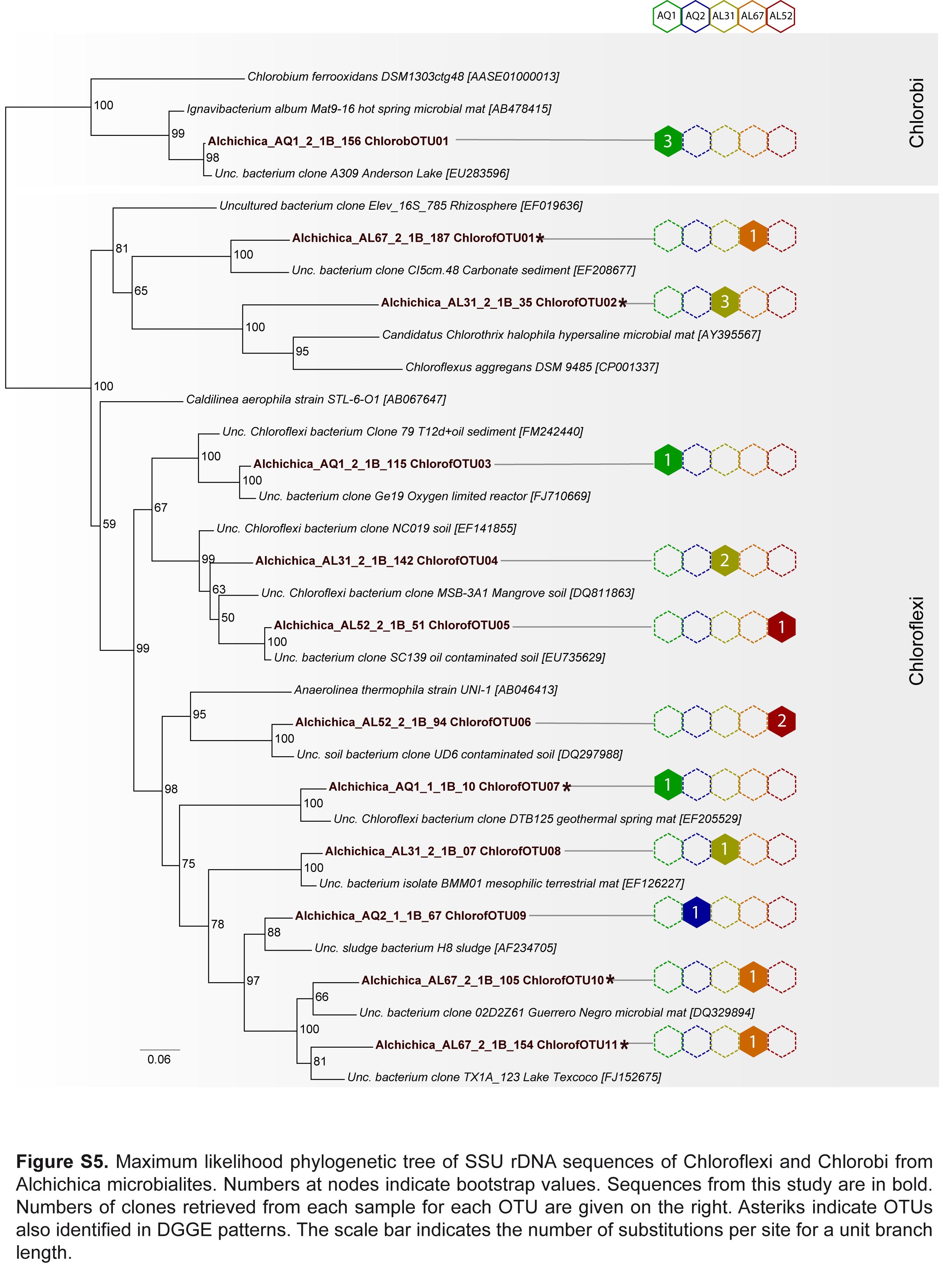

Supplement: Figure S5 — Maximum likelihood phylogenetic tree of SSU rDNA sequences of Chloroflexi and Chlorobi from Alchichica microbialites. Numbers at nodes indicate bootstrap values. Sequences from this study are in bold. Numbers of clones retrieved from each sample for each OTU are given on the right. Asterisks indicate OTUs also identified in DGGE patterns. The scale bar indicates the number of substitutions per site for a unit branch length. (TIF) [file pone.0028767.s005.tif]

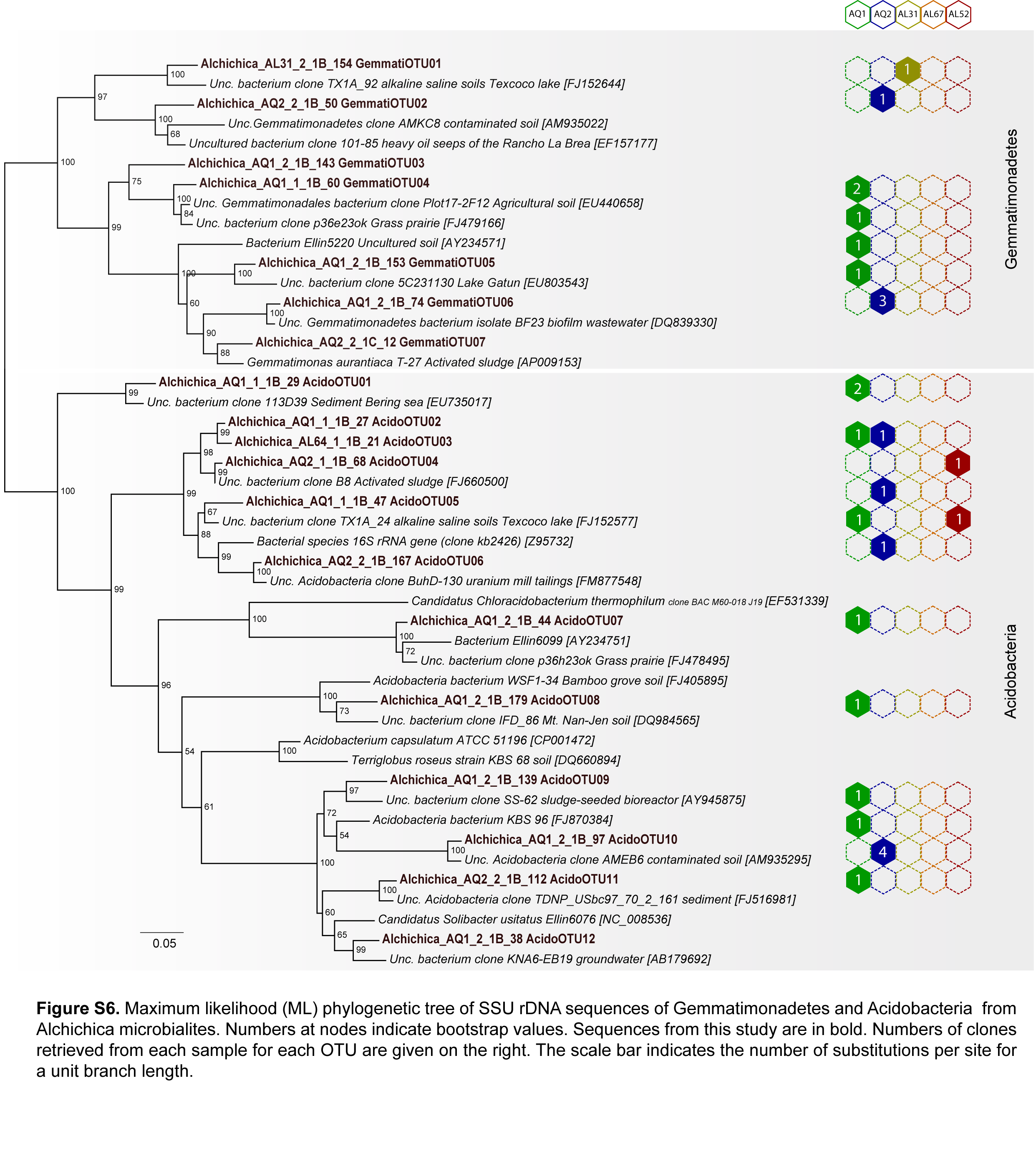

Supplement: Figure S6 — Maximum likelihood (ML) phylogenetic tree of SSU rDNA sequences of Gemmatimonadetes and Acidobacteria from Alchichica microbialites. Numbers at nodes indicate bootstrap values. Sequences from this study are in bold. Numbers of clones retrieved from each sample for each OTU are given on the right. The scale bar indicates the number of substitutions per site for a unit branch length. (TIF) [file pone.0028767.s006.tif]

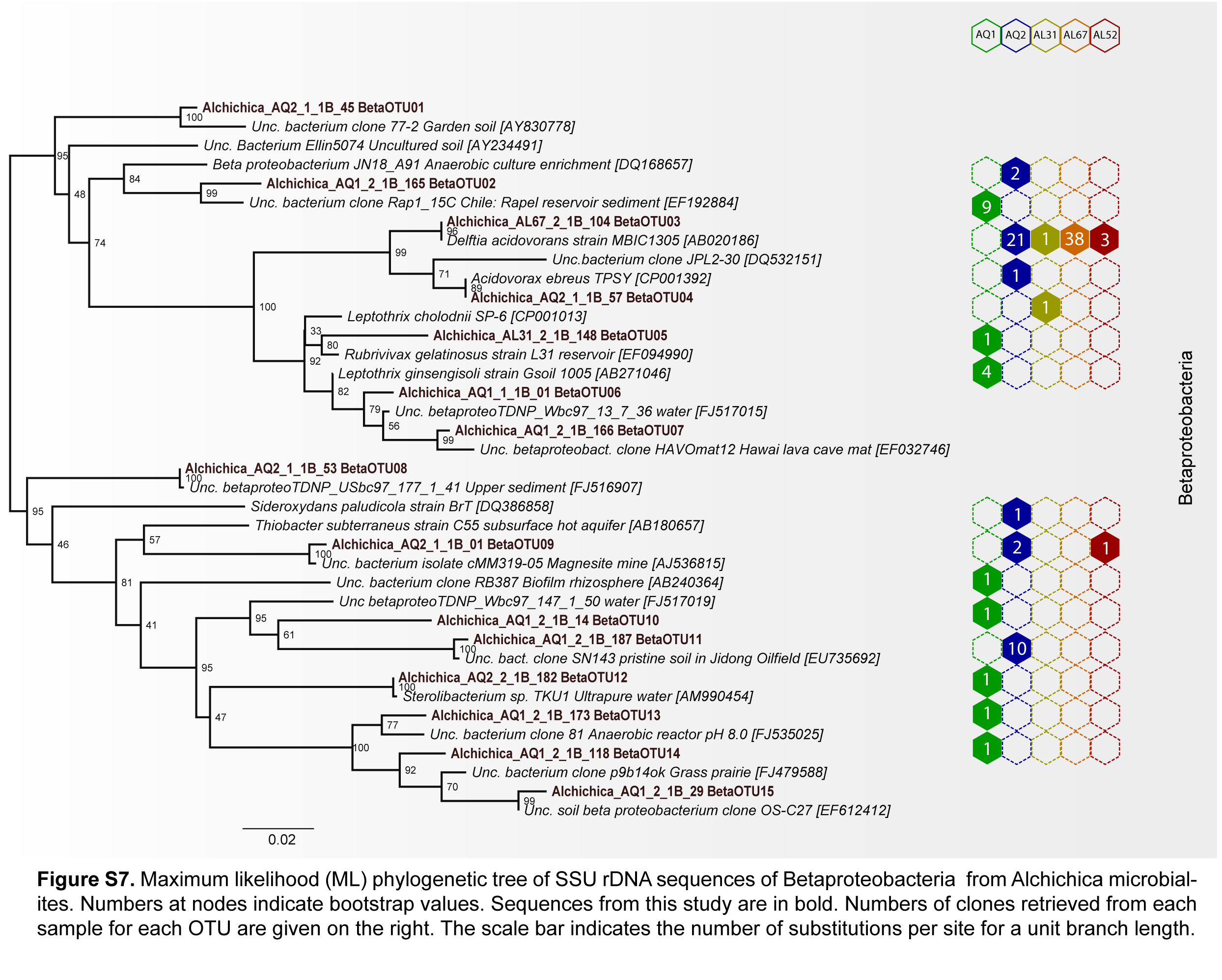

Supplement: Figure S7 — Maximum likelihood (ML) phylogenetic tree of SSU rDNA sequences of Betaproteobacteria from Alchichica microbialites. Numbers at nodes indicate bootstrap values. Sequences from this study are in bold. Numbers of clones retrieved from each sample for each OTU are given on the right. The scale bar indicates the number of substitutions per site for a unit branch length. (TIF) [file pone.0028767.s007.tif]

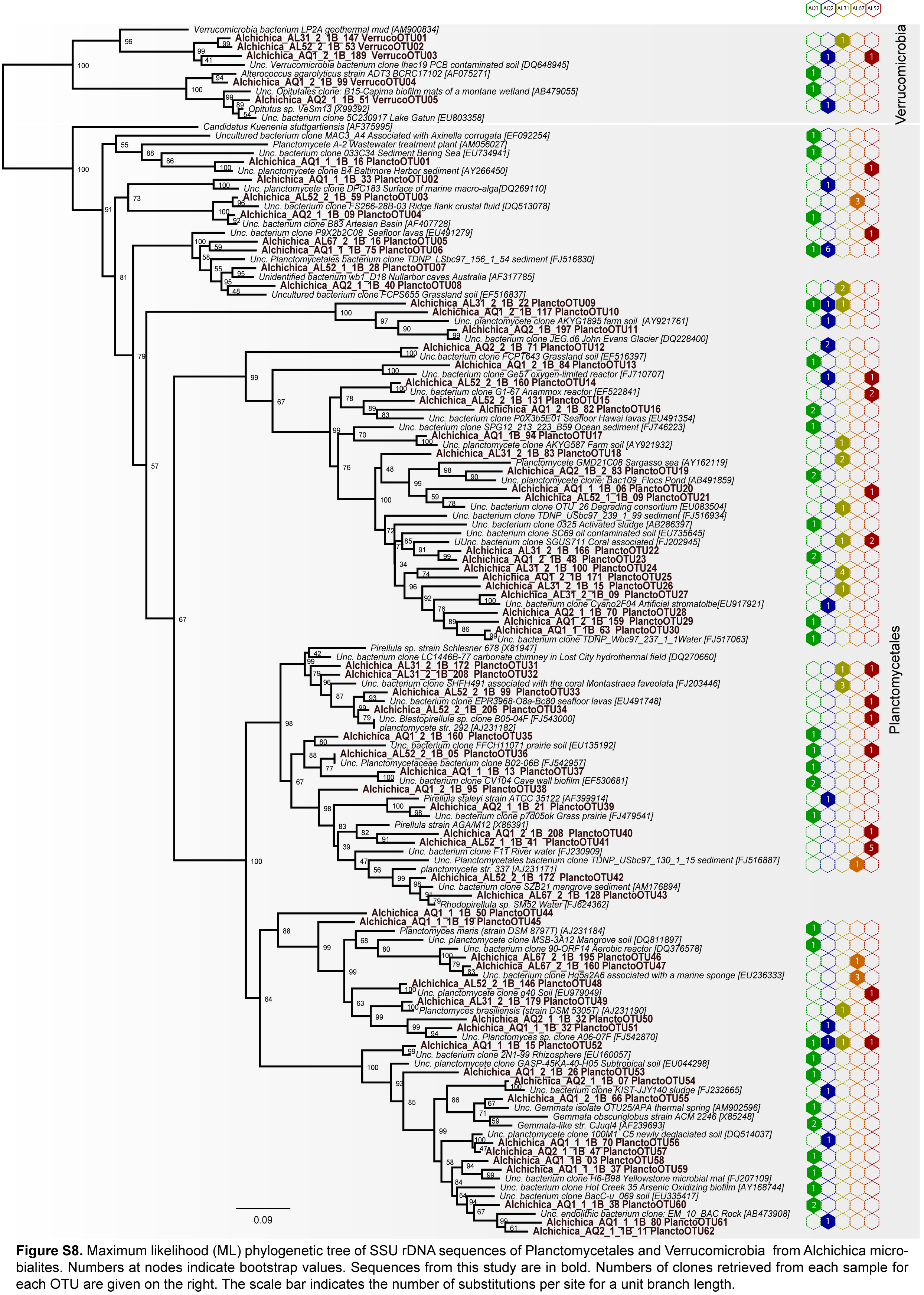

Supplement: Figure S8 — Maximum likelihood (ML) phylogenetic tree of SSU rDNA sequences of Planctomycetales and Verrucomicrobia from Alchichica microbialites. Numbers at nodes indicate bootstrap values. Sequences from this study are in bold. Numbers of clones retrieved from each sample for each OTU are given on the right. The scale bar indicates the number of substitutions per site for a unit branch length. (TIF) [file pone.0028767.s008.tif]

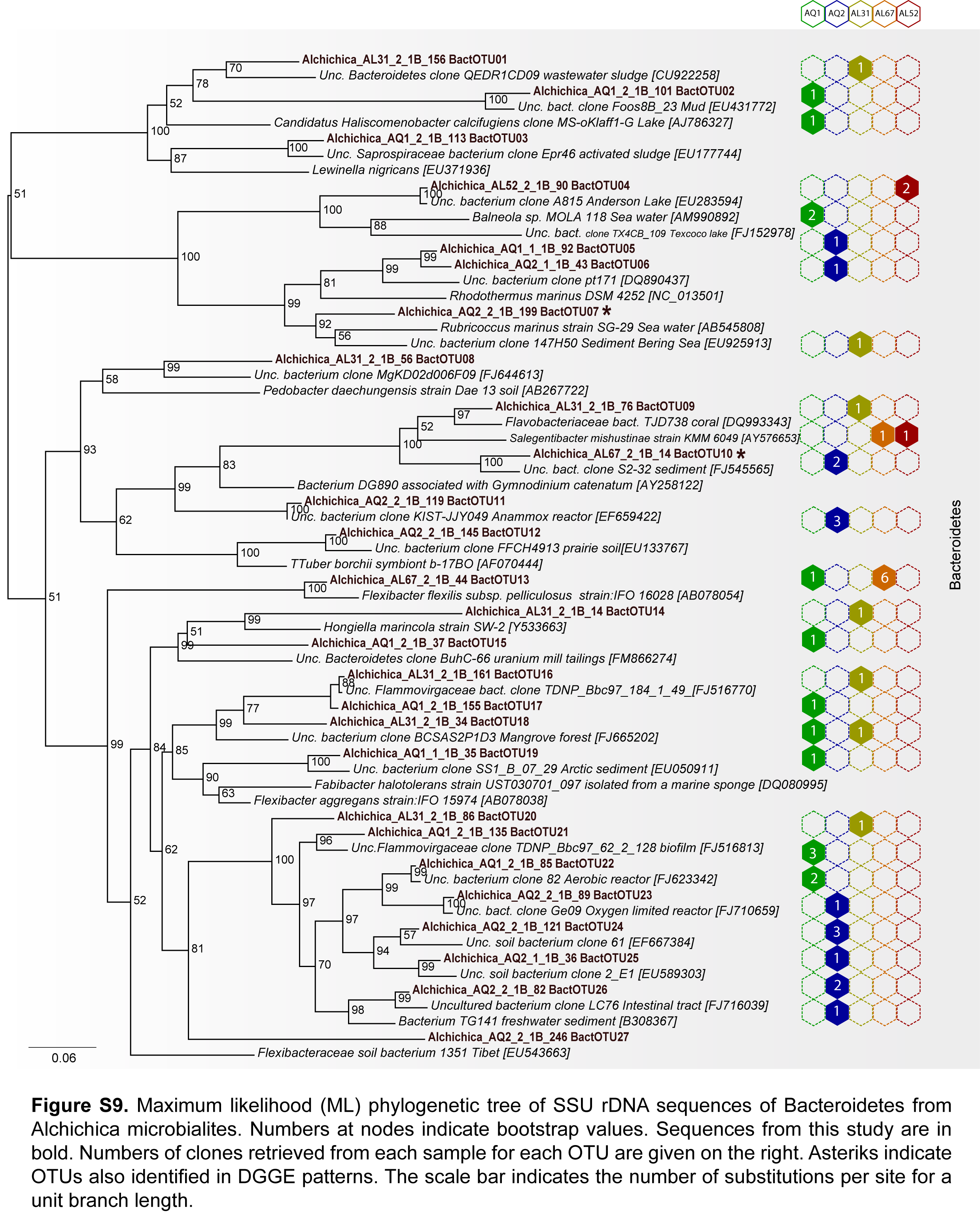

Supplement: Figure S9 — Maximum likelihood (ML) phylogenetic tree of SSU rDNA sequences of Bacteroidetes from Alchichica microbialites. Numbers at nodes indicate bootstrap values. Sequences from this study are in bold. Numbers of clones retrieved from each sample for each OTU are given on the right. Asterisks indicate OTUs also identified in DGGE patterns. The scale bar indicates the number of substitutions per site for a unit branch length. (TIF) [file pone.0028767.s009.tif]

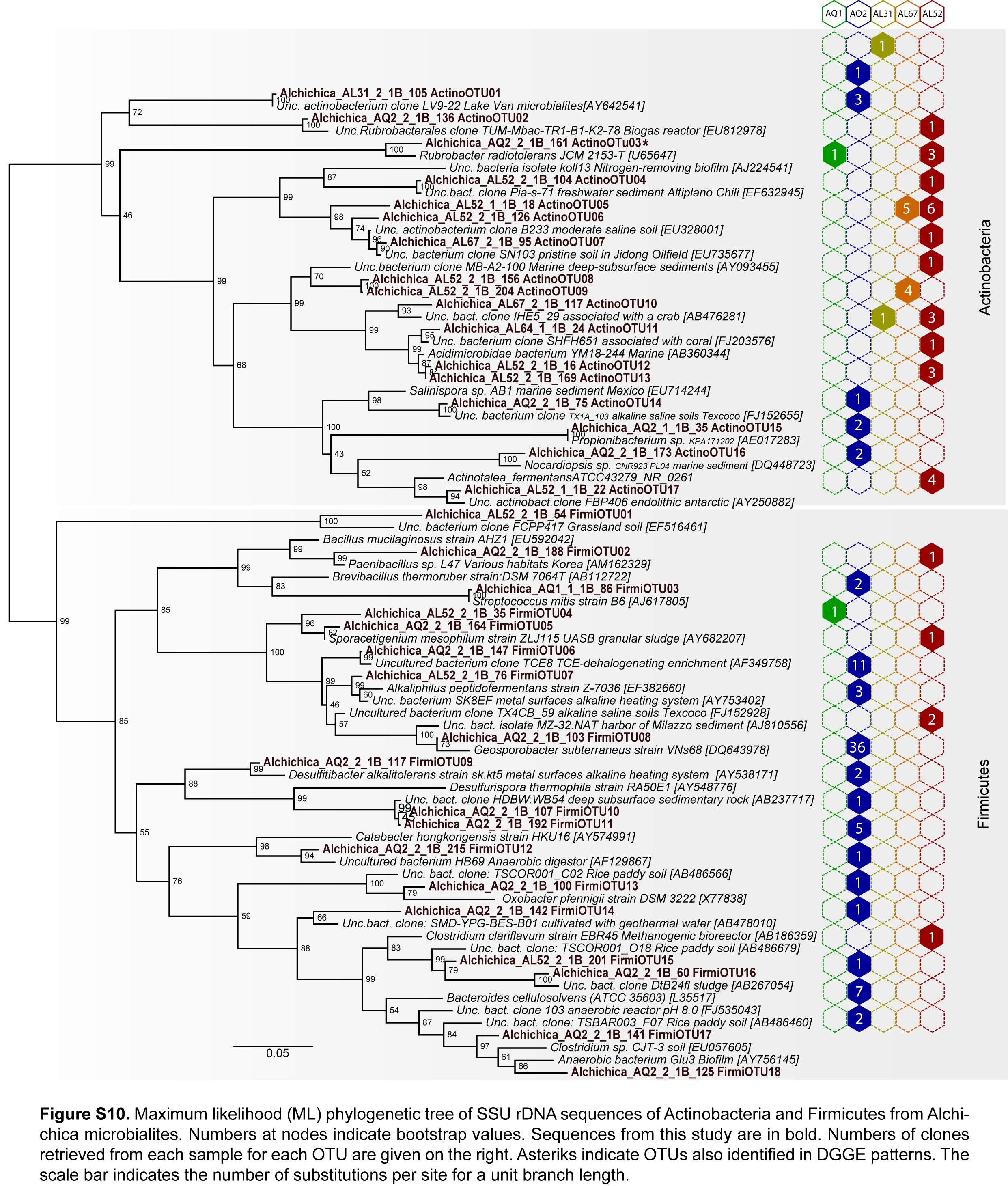

Supplement: Figure S10 — Maximum likelihood (ML) phylogenetic tree of SSU rDNA sequences of Actinobacteria and Firmicutes from Alchichica microbialites. Numbers at nodes indicate bootstrap values. Sequences from this study are in bold. Numbers of clones retrieved from each sample for each OTU are given on the right. Asterisks indicate OTUs also identified in DGGE patterns. The scale bar indicates the number of substitutions per site for a unit branch length. (TIF) [file pone.0028767.s010.tif]

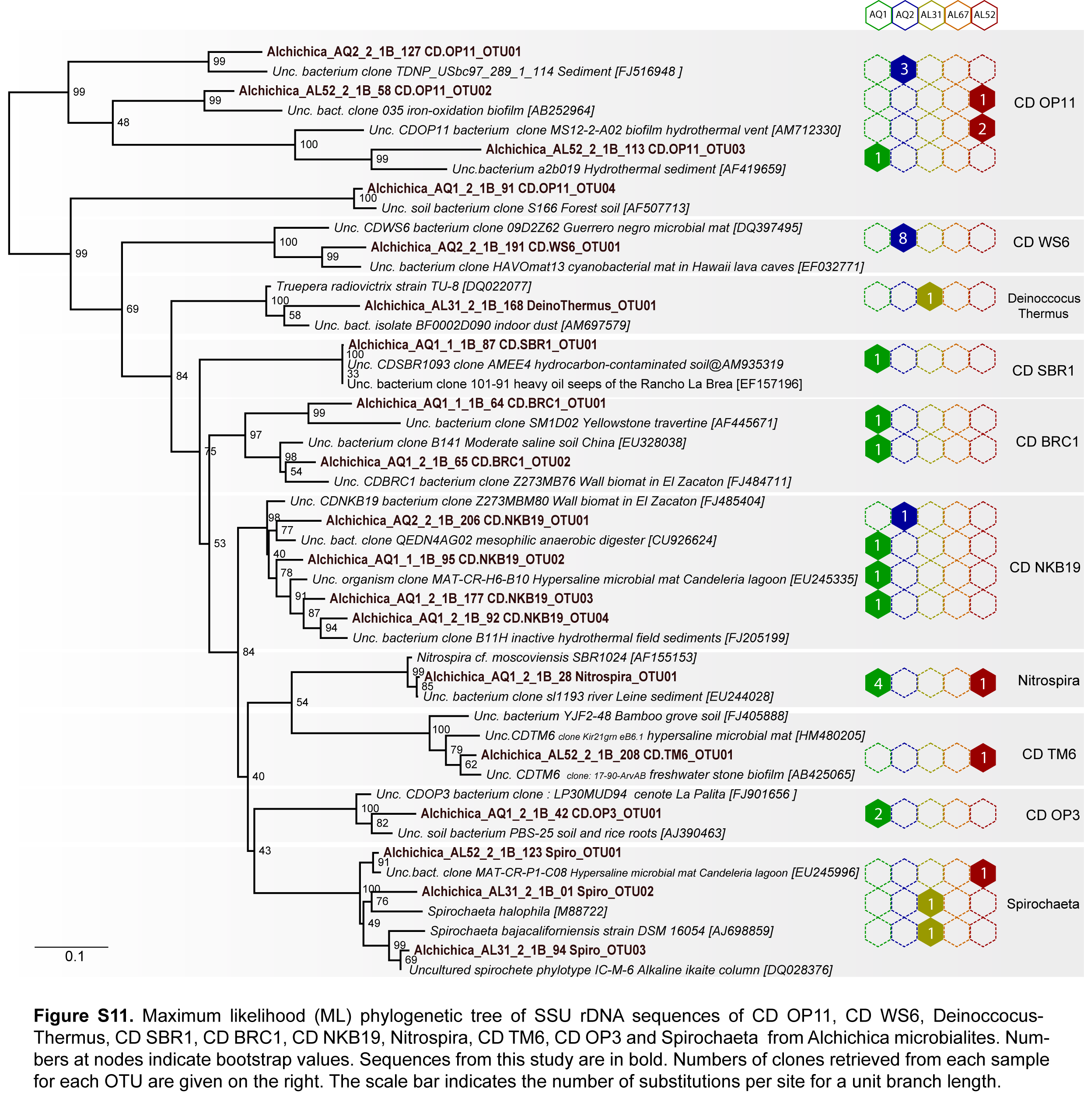

Supplement: Figure S11 — Maximum likelihood (ML) phylogenetic tree of SSU rDNA sequences of CD OP11, CD WS6, Deinoccocus-Thermus, CD SBR1, CD BRC1, CD NKB19, Nitrospira, CD TM6, CD OP3 and Spirochaeta from Alchichica microbialites. Numbers at nodes indicate bootstrap values. Sequences from this study are in bold. Numbers of clones retrieved from each sample for each OTU are given on the right. The scale bar indicates the number of substitutions per site for a unit branch length. (TIF) [file pone.0028767.s011.tif]

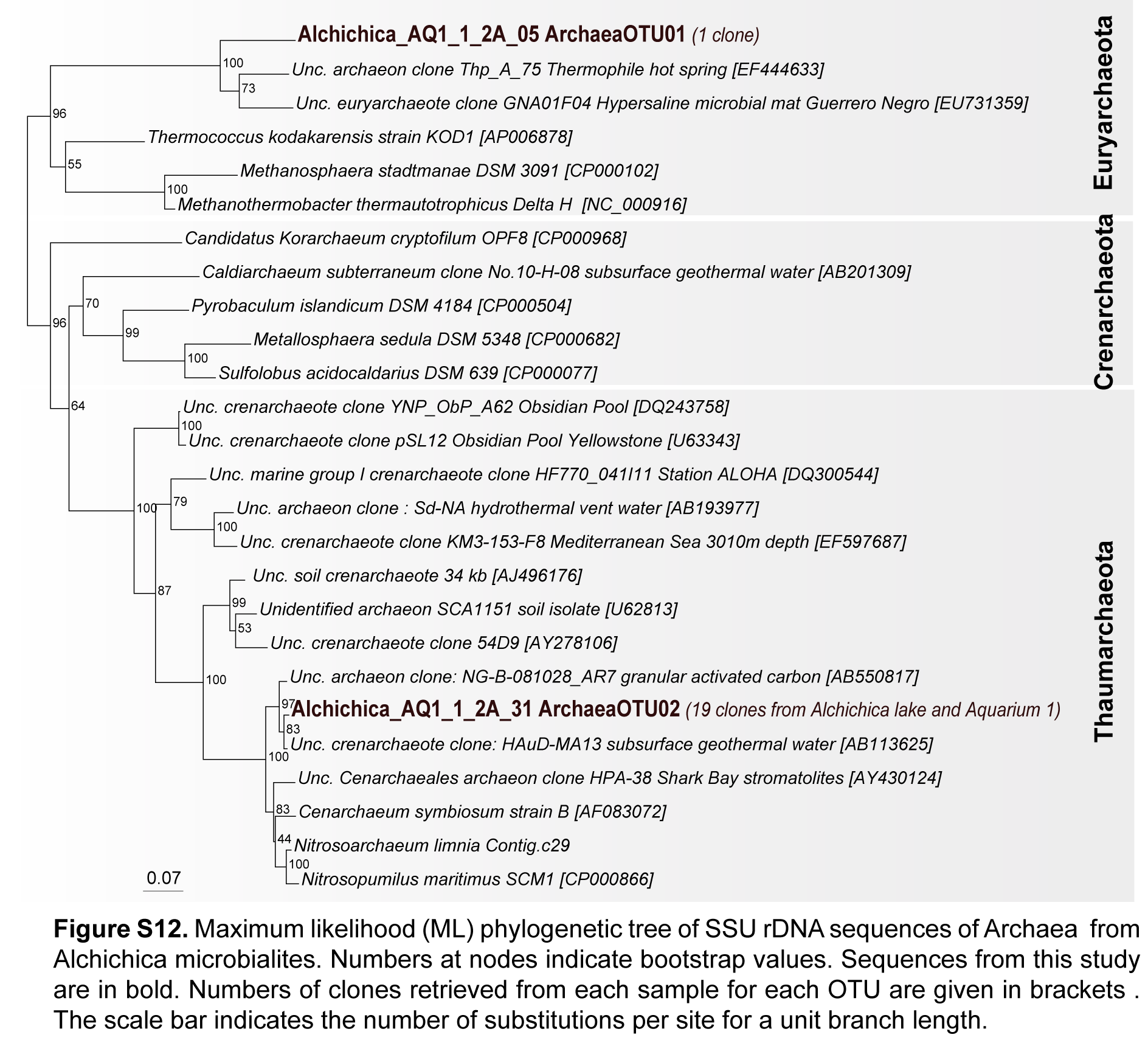

Supplement: Figure S12 — Maximum likelihood (ML) phylogenetic tree of SSU rDNA sequences of Archaea from Alchichica microbialites. Numbers at nodes indicate bootstrap values. Sequences from this study are in bold. Numbers of clones retrieved from each sample for each OTU are given in brackets. The scale bar indicates the number of substitutions per site for a unit branch length. (TIF) [file pone.0028767.s012.tif]

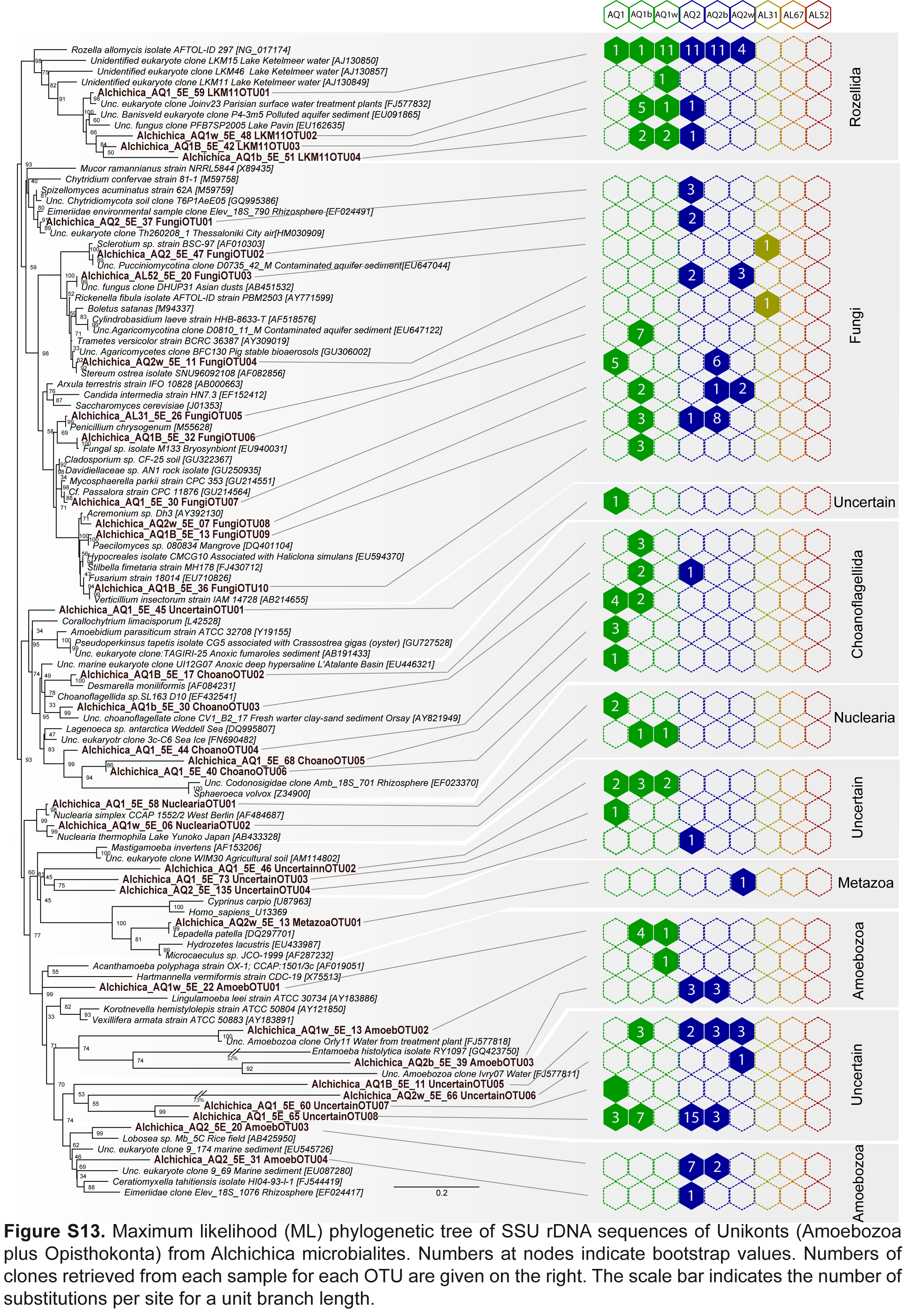

Supplement: Figure S13 — Maximum likelihood (ML) phylogenetic tree of SSU rDNA sequences of Unikonts (Amoebozoa plus Opisthokonta) from Alchichica microbialites. Numbers at nodes indicate bootstrap values. Numbers of clones retrieved from each sample for each OTU are given on the right. The scale bar indicates the number of substitutions per site for a unit branch length. (TIF) [file pone.0028767.s013.tif]

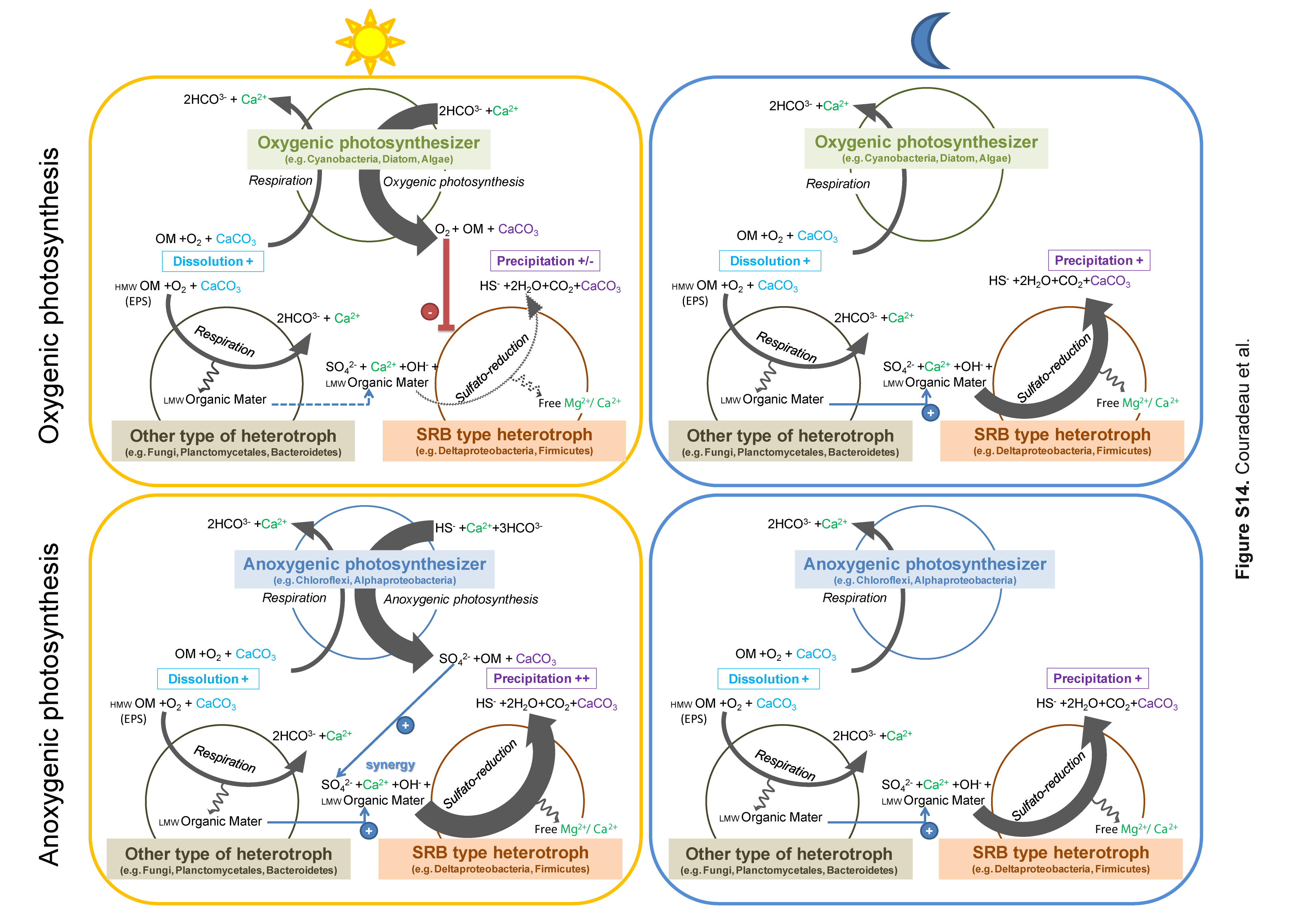

Supplement: Figure S14 — Hypothetical model of carbonate formation dynamics based on known metabolisms of microbial lineages detected in Alchichica microbialites. The panels represent the activities that would occur during day (left) and; night (right) in areas where oxygenic (upper panels) or anoxygenic (lower panels) photosynthesis predominates. (TIF) [file pone.0028767.s014.tif]
